# Bindel-PCR: a novel and convenient method for identifying CRISPR/Cas9-induced biallelic mutants through modified PCR using *Thermus aquaticus* DNA polymerase

**DOI:** 10.1038/s41598-019-46357-8

**Published:** 2019-07-09

**Authors:** Takayuki Sakurai, Akiko Kamiyoshi, Norio Takei, Satoshi Watanabe, Masahiro Sato, Takayuki Shindo

**Affiliations:** 1 Department of Life Innovation, Institute for Biomedical Sciences, Shinshu University, 3-1-1 Asahi, Matsumoto, Nagano 390-8621 Japan; 2 Department of Cardiovascular Research, School of Medicine, Shinshu University, 3-1-1 Asahi, Matsumoto, Nagano 390-8621 Japan; 3 Department of Molecular Therapeutics, Center for Food and Medical Innovation, Institute for the Promotion of Business-Regional Collaboration, Hokkaido University, Kita-21 Nishi-11, Kita-ku, Sapporo 001-0021 Japan; 4 Animal Genome Research Unit, Division of Animal Science, National Institute of Agrobiological Sciences, Ibaraki, 305-8602 Japan; 5 Section of Gene Expression Regulation, Frontier Science Research Center, Kagoshima University, 8-35-1 Sakuragaoka, Kagoshima, Kagoshima 890-8544 Japan

**Keywords:** Genetic engineering, Lab-on-a-chip

## Abstract

We developed a novel and convenient method for rapidly identifying CRISPR/Cas9-based genome-edited biallelic knockout (KO) cells/individuals carrying insertions or deletions of a few nucleotides (indels) by performing PCR on genomic DNA samples under stringent conditions and low MgCl_2_ concentrations. The biallelic KO samples can be judged as ‘negative’ under these conditions. The sense primer corresponds to the sequence recognised by guide RNA and subsequently cleaved by Cas9 immediately upstream of a target gene’s proto-spacer adjacent motif (PAM), and the reverse primer corresponds to the sequence ~200 bp downstream from the PAM. PCR performed using this primer set under standard MgCl_2_ concentrations (1.5–2.5 mM) should generate PCR products derived from both mutated and unedited alleles, whereas PCR performed using lower MgCl_2_ concentrations (0.8–2 mM) should yield products derived from unedited alleles. This enables high-throughput screening of biallelic mutants among cells/embryos having ≥1 indels at a region within 5 bp upstream of the PAM (where more than 94% of indels are known to appear). We performed proof-of-principle analyses of this novel approach using genome-edited *Et1, Tyr, Ramp1, Ramp3*, and *Rosa26* mouse samples carrying various types of indels, and demonstrate that this new technique allows rapid identification of biallelic KO mutants among samples carrying various types of indels and mosaic mutations with 100% accuracy. We name this system detection of biallelic KO mutants harbouring indels using PCR (Bindel-PCR).

## Introduction

Advances in the development of genome-editing systems, such as zinc-finger nuclease (ZFN), transcription activator-like effector nucleases (TALENs), and clustered regularly interspaced short palindromic repeats (CRISPR)/CRISPR-associated protein-9 nuclease (Cas9) (CRISPR/Cas9), provide breakthroughs in life science-related research^1–3^. The CRISPR/Cas9 system is now recognised as one of the most powerful tools for engineering genes of interest; this is because of the simplicity of the system and the high specificity it provides in targeted genome engineering. CRISPR/Cas9-based mutation induction commences with specific cleavage of double-stranded DNA 3–5 bp upstream of the proto-spacer adjacent motif (PAM) in a target locus^4,5^. The resulting cleavage can be repaired through homology-directed repair (HDR) (which requires homologous recombination with a homology arm), non-homologous end-joining (NHEJ) [which involves no homology or only 1–3 nucleotides (nt) of homology at the junction]^6^, or microhomology-mediated end-joining (MMEJ) [which involves alignment of microhomologous sequences (1–4 nt) internal to the broken ends before joining]^7^. Consequently, a DNA fragment can be inserted into the target locus precisely through HDR, and this generates insertion/deletion mutations (‘indels’) through NHEJ and MMEJ^8^.

Currently, gene-edited mice can be created readily and with high efficiency through one-step generation, which involves microinjection or *in vitro* electroporation of embryos in the presence of CRISPR/Cas9 reagents^9,10^. However, together with the development of this process, the demand for selecting biallelic knockout (KO) mutants has been increasing, because phenotypic alteration resulting from the dysfunction of a gene of interest can be readily detected. Therefore, the development of a simple and rapid method for screening biallelic mutants is eagerly awaited.

Genome-edited organisms and samples such as cells/embryos obtained through HDR can be readily identified because their rapid detection is possible through PCR performed using primers corresponding to the specific sequence in the inserted DNA fragment and the use of restriction enzymes that can selectively recognise the resulting PCR products^11,12^. By comparison, it is more challenging to identify organisms carrying indels generated through the NHEJ- or MMEJ-based repair pathway, because PCR-based amplification of an area showing alteration of a few nucleotides frequently fails to distinguish this sequence from the wild-type (WT) sequence. To date, the T7 endonuclease 1 (T7E1)-based assay and the Surveyor enzyme mismatch cleavage assay have been most frequently used to scan for indels triggered by engineered nucleases^13^. These methods are based on the identification of heteroduplex DNA formed after melting and hybridizing mutant and WT alleles, and the methods exploit the use of enzymes that can cleave heteroduplex DNA at mismatches formed by single or multiple nucleotides. However, to find the biallelic mutants with indels, these approaches occasionally require an additional *in vitro* assay such as Cas9RNP cut assay, in which the WT alleles are cleaved by a Cas9/ribonuclear protein complex prepared through *in vitro* complex formation between guide RNA (gRNA) and Cas9 protein^14^. Furthermore, the sensitivity of the methods must be increased to detect the presence of samples carrying mosaic mutations. When numerous samples must be precisely genotyped, these processes are expensive, laborious, and frequently time-consuming.

Here, we present a rapid and convenient assay system, named detection of biallelic KO mutants harbouring indels using PCR (Bindel-PCR), for the identification of CRISPR/Cas9-induced indels by employing only a general PCR apparatus and *Thermus aquaticus*-derived PCR DNA polymerase [recombinant *Taq* (*rTaq*) DNA polymerase or HiDi DNA polymerase]. Bindel-PCR requires 2 rounds of PCR to detect biallelic editing events in both alleles. The first round of PCR was carried out under standard concentrations of Mg^2+^ (1.5–2.5 mM) for confirming successful amplification of a target region. The second round of PCR was performed on the first PCR products under low MgCl_2_ concentrations. Biallelic KO samples are considered negative under these conditions. We show proof-of-principle analyses of this novel approach using genome-edited *Et1, Tyr, Ramp1, Ramp3*, and *Rosa26* mouse samples carrying various types of indels.

## Results

### Survey of the sites frequently showing CRISPR/Cas9-induced indels and primer design used for Bindel-PCR

**Figure 1:**
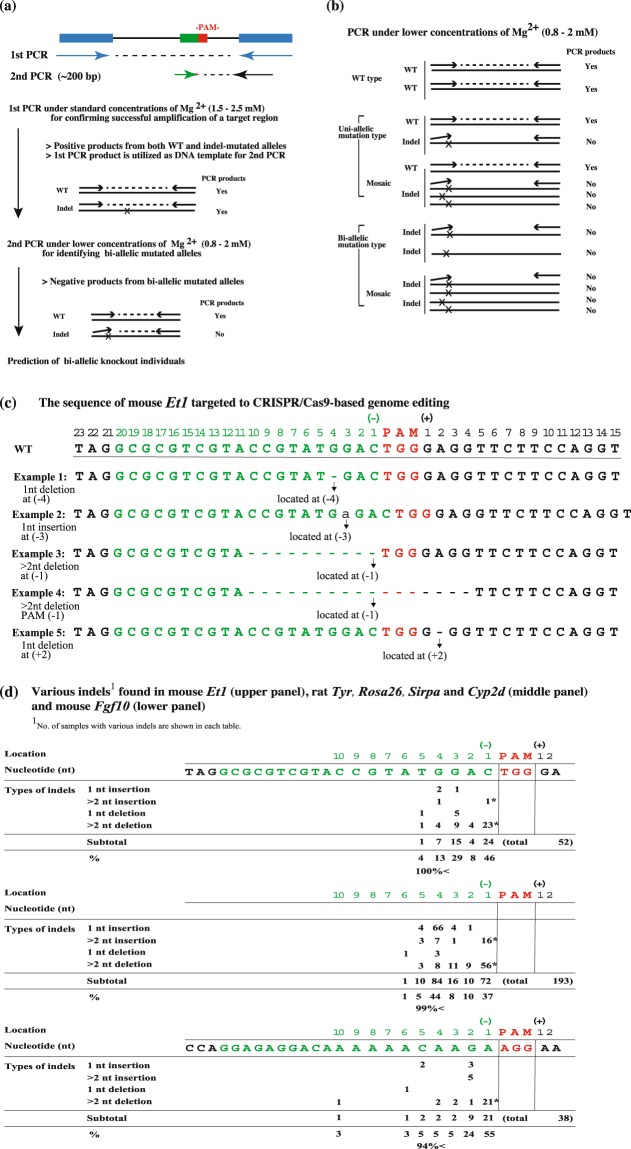
Principle of Bindel-PCR for identifying CRISPR/Cas9-induced biallelic mutants. (**a**) Outline of Bindel-PCR. 1st PCR is carried out under standard concentrations of Mg^2+^ (1.5–2.5 mM) for confirming successful amplification of a target region. For the 2nd PCR, the sense primer was set corresponding to the sequence that is recognised by gRNA and cleaved by Cas9 immediately upstream of the PAM of the target gene, whereas the reverse primer was set on a downstream sequence distant from the PAM. PCR performed using this primer set and the 1^st^ PCR products as template under standard conditions should generate PCR products (~200 bp) derived from both indel-mutated alleles and unedited alleles. Contrastingly, when the concentration of MgCl_2_ used for PCR is lowered, annealing between a primer and the mutated target sequence becomes unstable, and under this condition, PCR products derived from unedited alleles, such as WT allele, should be generated preferentially. Mg^2+^ is recognised as not only a cofactor for DNA polymerase activity, but also a promoter of complex formation between primers and DNA template^26^. Blue boxes, sequences recognised by forward and reverse primers used for 1st PCR; green box, sequence recognised by crRNA; red box, PAM sequence. (**b**) Flowchart of detection of biallelic KO mutants by using Bindel-PCR. (**c**) The evaluation rule for indels appearing in the target sequence is shown using mouse *Et1* gRNA-recognizing nt region as an example. Green nt correspond to the sequence recognised by gRNA-*Et1*. Red nt correspond to the PAM sequence. nt −1 to –23 correspond to the 5′ upstream region of the PAM. nt +1 to +15 correspond to the 3′ downstream region of the PAM. Arrows indicate the site of indels classified. WT, wild-type. (**d**) Analyses of sites frequently showing CRISPR/Cas9-induced indels. Data shown are from our previous study (Sakurai *et al*., unpublished data) on mouse *Et1* and from other studies [Yoshimi *et al*. (2016) for rat *Tyr, Rosa26, Sirpa*, and *Cyp2d* and Hashimoto and Takemoto (2015) for mouse *Fgf10*]. *Includes the samples with indels at nt (−1) as well as those with indels including a part of the PAM. *Et1*, endothelin-1; *Tyr*, tyrosinase; *Rosa26*, Gt(ROSA)26S; *Sirpa*, signal regulatory protein-α; *Cyp2d*, cytochrome P450, family 2, subfamily d; *Fgf10*, fibroblast growth factor-10.

Bindel-PCR principle and flowchart are shown in Fig. 1a,b and described in its corresponding figure legend. To design the primers used for Bindel-PCR for detecting biallelic mutants, we first scanned for the sites that had been frequently genome-edited after recognition by gRNA and subsequent cleavage by Cas9; for this, we used data obtained from our recent study [for mouse endothelin-1 gene (*Et1*) (Sakurai *et al*., unpublished data)] and other studies [for rat tyrosinase gene (*Tyr*), *Rosa26* (Gt(ROSA)26S), signal regulatory protein-α gene (*Sirpa*), and cytochrome P450 family 2 subfamily D gene (*Cyp2d*)^15^, and for mouse fibroblast growth factor-10 gene (*Fgf10*)^11^]. In Fig. 1c, various types of indels in mouse *Et1* are shown as examples. The gRNAs used were designed to correspond to the 20-bp sequence (shown by green colour) preceding the PAM. The nt immediately before the PAM was designated as nt (−1), and the nt immediately after the PAM as nt (+1). For example, the sample with one nt deletion at nt (−4) (shown as Example 1 in Fig. 1c) can be shown as “1 nt deletion at nt (−4)”. The sample with one nt insertion at nt (−3) (shown as Example 2 in Fig. 1c) can be shown as “1 nt insertion at nt (−3)”. The sample with more than one nt at nt (−1) (shown as Example 3 in Fig. 1c) can be shown as “>2 nt deletion at nt (−1)”. The sample containing indels of >2 nt spanning the PAM (shown as Example 4 in Fig. 1c) was defined as PAM (−1), which was classified as >2 nt deletion at nt (−1). In Fig. 1d, data from the present study (for mouse *Et1*) and previous studies (for rat *Tyr*, *Rosa26*, *Sirpa*, and *Cyp2d* as well as mouse *Fgf10*) are provided in brief. The site cleaved by the CRISPR/Cas9 system is widely recognised to occur around 3 bp upstream of the PAM frequently^4,5^. Corresponding to this notion, the accumulated data indicated that indels occurred frequently at the sites [corresponding to nt (−6) to (−1)] before PAM (Fig. 1d). Notably, 97.6% of indels (100% for mouse *Et1*; 99% for rat *Tyr*, *Rosa26*, *Sirpa*, and *Cyp2d*; 94% for mouse *Fgf10*) were generated at nt (−5) to PAM (−1). Based on these results, a region at nt (−5) to PAM (−1) of a target gene was considered as an important target site for setting Bindel-PCR primers.

### Determination of optimal conditions for Bindel-PCR performed using *rTaq* DNA polymerase

**Figure 2:**
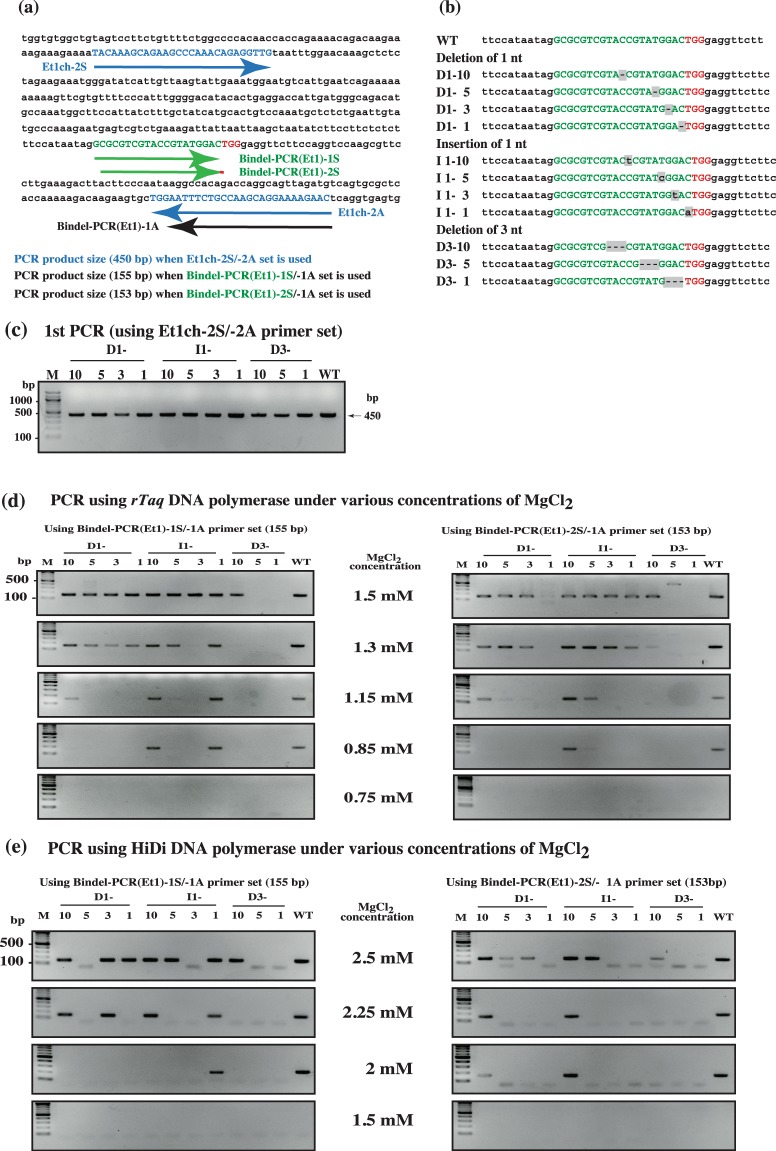
Bindel-PCR to identify biallelic KO mutants for *Et1*. (**a**) nt sequence in mouse *Et1* locus. The location of sequences recognised by gRNA and PCR primers is shown. nt in blue correspond to forward and reverse primers used for 1^st^ PCR. Green nt correspond to the sequence recognised by gRNA-*Et1*. Red nt correspond to the PAM. Expected sizes of PCR products obtained using different primer sets are shown at the bottom. (**b**) nt sequences in mouse *Et1* locus that are recognised by gRNA-*Et1*. Various types of indels found in a series of *Et1* plasmids are shown. nt in green correspond to the sequence recognised by gRNA-*Et1*. nt in red correspond to the PAM sequence. Shading indicates modification of Nt. Dashed lines indicate Nt deletion. (**c**) Gel electrophoretic pattern of PCR products (450 bp) obtained through 1^st^ PCR performed using Et1ch-2S/−2A primer set and a series of Et1 plasmids harbouring various types of indels as template. M, 100-bp-ladder markers. WT, wild-type. The images of these full-length gels are presented in Supplementary Fig. S2c. (**d**) (**e**) Gel electrophoretic pattern of products from the 2^nd^ PCR (corresponding to *Et1* sequence recognised by gRNA-*Et1*) obtained after performing PCR on a series of *Et1* plasmids [shown in (**b**) and (**c**)] under reduced concentrations of MgCl_2_. Representative results from assays performed using *rTaq* DNA polymerase are shown in (**d**). Left panel, 2^nd^ PCR products (155 bp) obtained using Bindel-PCR(Et1)-1S/-1A primer set; right panel, 2^nd^ PCR products (153 bp) obtained using Bindel-PCR(Et1)-2S/-1A primer set. Representative results from assays performed using HiDi DNA polymerase are shown in (**e**). Left panel, products from the 2^nd^ PCR (155 bp) obtained using Bindel-PCR(Et1)-1S/-1A primer set; right panel, 2^nd^ PCR products (153 bp) obtained using Bindel-PCR(Et1)-2S/-1A primer set. M, 100-bp-ladder markers; WT, wild-type. The images of these full-length gels are presented in Supplementary Fig. S2d,e.

Based on the above findings that indels occurred more frequently within an area of 5 bp 5′ upstream of the PAM, we decided to use the samples derived from F0 offspring (Sakurai *et al*., unpublished data) with CRISPR/Cas9 based genome-edited *Et1* gene for exploring optimal conditions of Bindel-PCR. We used a PCR primer set (Et1ch-2S/-2A) (Fig. 2a) to amplify a portion of *Et1* locus spanning a sequence recognised by gRNA-*Et1* (corresponding to the sequence shown by green colour in Fig. 2a), and employed a series of *Et1* plasmids carrying various types of indels (Fig. 2b). For example, the plasmids containing inserts with 1 nt deletion at nt (−10), (−5), (−3), and (−1) were named D1-10, D1-5, D1-3, and D1-1, respectively. Similarly, plasmids containing inserts with 1 nt insertion at nt (−10), (−5), (−3), and (−1) were named I1-10, I1-5, I1-3, and I1-1, and those with 3 nt deletion at nt (−10), (−5), and (−1) were named D3-10, D3-5, and D3-1, respectively. Next, using these plasmids carrying various types of *Et1* indels, we explored optimal conditions for Bindel-PCR to identify biallelic indels using *rTaq* DNA polymerase. For this, a sense PCR primer [Bindel-PCR(Et1)1S: 5′-GCGCGTCGTACCGTATGGAC-3′] and a nested sense primer [Bindel-PCR(Et1)2S: 5′-CGCGTCGTACCGTATGGACT-3′] (Fig. 2a) were used. The melting temperature (Tm) of these primers ranged from ~67 °C to 70 °C. We selected the site recognised by the antisense primer [Bindel-PCR(Et1)1A: 5′-GTTCTTTTCCTGCTTGGCAGAAATT-3′: Tm, 70 °C] approximately 200 bp away from the positions recognised by these sense primers (Fig. 2a). In Bindel-PCR, the mutated samples failed to generate amplified products. To avoid the formation of false-positive PCR products, the portion of *Et1* spanning the sequence recognised by gRNA-*Et1* was first amplified as the 1^st^ PCR (Fig. 2c). The resultant PCR products were next subject to nested PCR to ascertain the optimal conditions that allow successful amplification of a target sequence from the WT but not mutated allele.

First, the 1^st^ PCR was performed using plasmids (5 ng/tube) containing various types of indels at *Et1* locus (PCR protocol: 95 °C for 1 min, 35 cycles of 95 °C for 15 s, 68 °C for 10 s, and 72 °C for 40 s) in a PCR buffer [10 mM Tris (pH 8.3), 50 mM KCl, 200 μM dNTPs, and 1.5 mM MgCl_2_] containing *rTaq* DNA polymerase (0.2 U/25 μL) and Et1ch-2S/-2A primers. The expected 450-bp amplification band was obtained from all the plasmid samples used (Fig. 2c).

Next, as the 2^nd^ PCR, nested PCR was performed using Bindel-PCR (Et1)-1S/-1A or Bindel-PCR (Et1)-2S/-1A primer set (Fig. 2a). We first tested the annealing temperature, various dNTP concentrations, 2-step PCR (shuttle PCR), and 3-step touchdown PCR with annealing at 72 °C (decreasing 1 °C per cycle to 61 °C), which are considered as a simple and rapid means to optimise PCR^21^, for the potential establishment of Bindel-PCR, but failed to obtain a reproducible condition for Bindel PCR (data not shown). Consequently, we found that Mg^2+^ concentration and the location of primers as one of the critical factors for Bindel-PCR. Thus, the 1^st^ PCR products (0.5–1 ng) (Fig. 2c) were subject to PCR under these conditions: 95 °C for 1 min, 40–45 cycles of 95 °C for 15 s, 68 °C for 10 s, and 72 °C for 40 s; the annealing temperature of 68 °C was employed according to the Tm (~70 °C) of the primers used (Fig. 2d). PCR was repeated thrice for each condition to confirm reproducibility. The amplification efficiency of a target sequence tended to decrease with a decrease in Mg^2+^ concentration, but the results clearly indicated that the inability to detect the sequence carrying indels depended on the Mg^2+^ concentration (Fig. 2d). For example, no amplification product was obtained from either the WT or mutated allele when PCR was performed, using Bindel-PCR(Et1)-1S/-1A primer set (Fig. 2d), in the presence of 0.75 mM MgCl_2_; by contrast, PCR in the presence of 1.5 mM MgCl_2_ yielded amplification products from these samples and from samples (D3-5 and D3-1) carrying a 3-bp deletion (Fig. 2d, left panel), and PCR [using Bindel-PCR(Et1)-1S/-1A primer set] in the presence of 0.85 mM MgCl_2_ clearly yielded products derived only from the samples WT, I1-10, and I1-1 (Fig. 2d, left panel). Sample I1-1 was positively identified here probably because it contained 1 nt added to the sequence type of nt (−1) (Fig. 2a,b). Therefore, Bindel-PCR(Et1)-2S/-1A primer set (Fig. 2a) was adopted. The PCR-amplification mode used was the same as that used for amplifying various samples (Fig. 2b). The results showed that D1-10 reacted weakly under this PCR condition; as expected, the sample I1-1 was negative, but sample I1-10 was still positive. These results indicate that it is possible to identify biallelic mutants by Bindel-PCR among the samples with indels having 1 ≥ nt deletion and insertion at nt (−5) to PAM (−1) of a target gene. However, when these results were combined with the data shown in Fig. 1d, the frequency of detection of samples carrying indels, as exemplified by the samples I1-10 and D1-10, was found to be very low. Based on these results, we determined the optimal PCR conditions that enable detection of almost all the samples carrying indels at nt (−5) to PAM (−1) of a target gene (coverage of indels ranged 94–100%; see Fig. 1d).

### Determination of optimal conditions for HiDi DNA polymerase-based Bindel-PCR

*T. aquaticus*-derived HiDi DNA polymerase is known to discriminate between mismatched primers^16^. To investigate whether this enzyme can be used for selecting biallelic KO samples harbouring indels, we examined the optimal conditions for HiDi DNA polymerase-based Bindel-PCR (Fig. 2e). The 1^st^ PCR was the same as that shown in Fig. 2c, and the PCR products generated by the 1^st^ PCR were used as templates (0.5–1 ng/tube) for the nested PCR. The nested PCR was performed in PCR solutions that were composed of 50 mM Tris (pH 9.2), 16 mM (NH_4_)_2_SO_4_, MgCl_2_ (ranging from 2.5 to 1.5 mM), 0.1% Tween-20, and 200 μM dNTPs and contained the primer set Bindel-PCR(Et1)-1S/-1A (or Bindel-PCR(Et1)-2S/-1A) and HiDi DNA polymerase (0.2 U/25 μL) (PCR protocol: 95 °C for 1 min, 40–45 cycles of 95 °C for 15 s, 68 °C for 10 s, and 72 °C for 40 s). PCR was performed thrice for each condition to confirm reproducibility. When HiDi DNA polymerase was used, the PCR-amplification efficiency of a target sequence tended to decrease with a decrease in Mg^2+^ concentration, indicating again that the inability to detect sequences carrying indels was dependent on the Mg^2+^ concentration. In the presence of 2 mM MgCl_2_, the reaction amplified none of the samples except WT and I1-1 (Fig. 2e, left panel). The sample I1-1 showed positive reaction probably because of the same reason it was positive when *rTaq* DNA polymerase was employed (preceding subsection; Fig. 2d, left panel). Notably, with Bindel-PCR(Et1)-2S/-1A primer set, the samples except for WT, D1-10, and I1-10 were PCR negative, when MgCl_2_ concentrations ranged between 2.25 and 2 mM (Fig. 2e, right panel). This might be solely the result of the ability of HiDi DNA polymerase^16^ to discriminate between mismatched primers or a result of the combined use of the polymerase and Bindel-PCR(Et1)-2S primer. PCR performed in the presence of 1.5 mM MgCl_2_ produced no positive products, not even from the WT sample (Fig. 2e, right panel). Notably, under the stringent conditions (where Mg^2+^ concentration was lowered), HiDi DNA polymerase tended to show superior performance relative to *rTaq* DNA polymerase in terms of the inability to amplify samples harbouring indels. Thus, we successfully determined the optimal conditions that lead to the inability to amplify almost all samples carrying indels at the PAM (−10) site and its downstream portion.

### Evaluating sensitivity of Bindel-PCR for identifying biallelic mutants among mixed samples containing various indels through comparison with T7 endonuclease 1 (T7E1) assay and *polyacrylamide gel* electrophoresis (PAGE)

**Figure 3:**
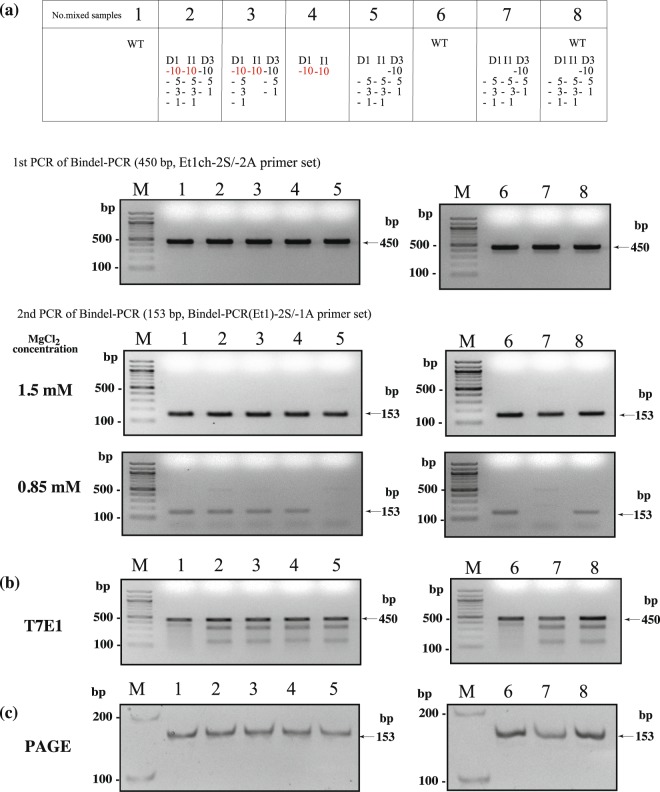
Analysis of sensitivity of Bindel-PCR for identifying biallelic mutants among mixed samples containing various indels. (**a**) Bindel-PCR of mixed *Et1* indel samples. Top row: the list of mixed samples containing plasmids carrying various indels shown in Fig. 2b. Both D1-10 and I1-10 (all of which is shown by red colour) are the samples that are proven not to detect the indels after analysis using Bindel-PCR (see Fig. 2d,e). WT, wild-type genomic DNA used as a positive control; M, 100-bp-ladder markers. Upper panel: 1st PCR (450 bp) of Bindel-PCR using Et1ch-2S/-2A primer set. Middle panel: 2nd PCR (153 bp) of 1st Bindel PCR products (upper panel) under 1.5 mM MgCl_2_ concentration and using Bindel-PCR(Et1)-2S/-1A primer set. Lower panel: 2nd PCR (153 bp) of 1st Bindel PCR products (upper panel) under 0.85 mM MgCl_2_ concentration and using Bindel-PCR(Et1)-2S/-1A primer set. (**b**) The results of T7E1 assay of 1st Bindel-PCR products [see upper panel of (a)]. (**c**) The results of 5% PAGE of 2nd Bindel-PCR products [see middle panel of (a)]. The images of these full-length gels are presented in Supplementary Figs S3a–c.

We performed Bindel-PCR using samples mixed with various types of plasmids (shown in Fig. 2b). In the upper panel of Fig. 3a, the content of plasmid in each mixed sample is shown. For example, sample numbered 1 and 6 contain WT. Sample numbered 2 is mixed and contains D1 series (D1-10, −5, −3 and −1), I1 series (I1-10, −5, −3 and −1) and D3 series (D3-10, −5 and −1). Simultaneously, T7E1 assay and PAGE were performed using the same samples used for Bindel-PCR. Identification of indels in the mouse *Et1* locus was based on the method shown in Fig. 2d,e. Bindel-PCR in the presence of 1.5 mM MgCl_2_ yielded clear PCR products in the samples numbered 5 and 7 (all of which lack D1-10 and L1-10), but failed in the presence of 0.85 mM MgCl_2_ (lower low of Fig. 3a), which appears to be consistent with the results shown in Fig. 2d,e. These results indicate that Bindel-PCR can confer identification of biallelic mutants among the mixed samples with various indels, at least at nt (−5) to PAM (−1) of a target gene. The results obtained after T7E1 assay are shown in Fig. 3b. The mixed samples numbered 1 and 6 were wild-type controls. As expected, they exhibited resistance against T7E1 cleavage. Notably, the cleavage pattern in the mixed samples numbered 5 and 7, which had been identified as biallelic mutation after Bindel-PCR-based analysis, was identical to those numbered 2, 3, 4, 7, and the WT sample. Consequently, this suggests that T7E1-based identification of biallelic mutation requires an additional *in vitro* assay (e.g., Cas9RNP cut assay). The results obtained after PAGE are shown in Fig. 3c. General PAGE/TBE system based analysis of the mixed samples revealed that there were no additional bands throughout the samples used. The mixed samples used in this study contain indels with 1 or 3 nt deletion or those with 1 nt insertion, these are also considered as “mosaic samples”. In this context, Bindel-PCR is useful to identify samples with biallelic mutations among the samples comprised by complicated mosaic mutations.

### Sensitivity of Bindel-PCR for identifying biallelic KO mice among genetically mosaic animals

**Figure 4:**
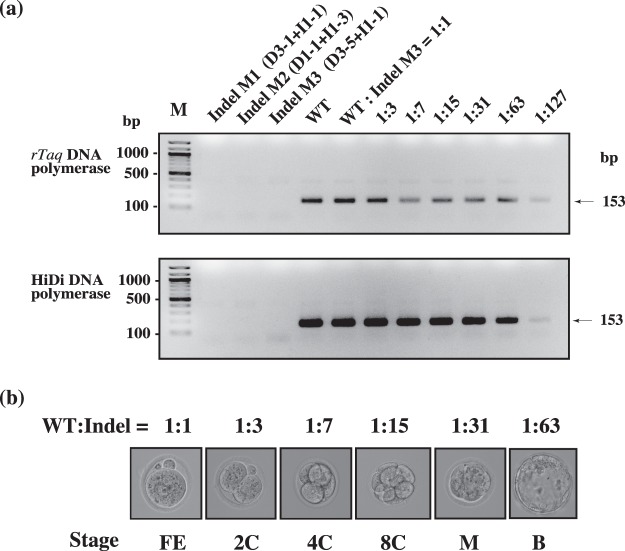
Analysis of sensitivity for detecting the presence of WT alleles in samples containing various ratios of mutant-type templates. (**a**) PCR of the 1^st^ PCR templates (shown in Fig. 2c) mixed at appropriate ratios. For example, Indel Mix 3 was prepared at the ratio of D3-5:I1-1 = 1:1. For preparing WT:Indel M3, WT DNA was added to Indel Mix 3 (D3-5:I1-1 = 1:1) at ratios of 1:1, 1:3, 1:7, 1:15, 1:31, 1:63, and 1:127. Upper panel: Results of 2^nd^ PCR performed using *rTaq* DNA polymerase and Bindel-PCR-2S/1A primer set in the presence of 0.85 mM MgCl_2_. Lower panel: Results of 2^nd^ PCR performed using HiDi DNA polymerase and Bindel-PCR(Et1)-2S/-1A primer set in the presence of 2 mM MgCl_2_. Each gel lane was loaded with 10 μL of PCR products. M, 100-bp-ladder markers. (**b**) WT:Indel samples prepared based on assuming that early mouse embryos are edited by CRISPR/Cas9-related components at these stages: zygote (FE, fertilised egg), 2-cell (2C), 4-cell (4C), 8-cell (8C), morula (M; comprising 16 cells), early blastocyst (B; 32 cells), and blastocyst (64–128 cells). The images of these full-length gels are presented in Supplementary Fig. S4a.

CRISPR/Cas9 is a powerful system for inducing genome editing in various organisms, but this technique frequently creates individuals carrying mosaic mutations, with various types of indels and WT sequences being included^17^. We considered it critical to test whether Bindel-PCR can detect WT alleles present in small numbers in a genome-edited animal carrying larger numbers of mutated alleles; a failure in this would lower the usability of Bindel-PCR. Here, we sought to measure the sensitivity for detecting the presence of WT alleles in mice carrying mosaic mutations. For this purpose, we used a set (Fig. 2b) that included various types of indels for *Et1*, and we prepared several samples in which certain types of indels were mixed at appropriate DNA:DNA ratios. For example, we prepared Indel Mix 1 (D1-5:I1-3 = 1:1), Indel Mix 2 (D1-3:D3-5 = 1:1), and Indel Mix 3 (D3-3:I1-3 = 1:1), and then added WT DNA to Indel Mix 3 at ratios of 1:1, 1:3, 1:7, 1:15, 1:31, 1:61, and 1:127; these were termed, respectively, WT:Indel Mix = 1:1, 1:3, 1:7, 1:15, 1:31, 1:61, and 1:127 (Fig. 4a). These ratios were tested based on the assumption of early mouse embryos being edited by CRISPR/Cas9-related components at these stages: zygote, 2-cell, 4-cell, 8-cell, morula (comprising 16 cells), early blastocyst (32 cells), and blastocyst (64–128 cells) (Fig. 4b). Each WT:Indel Mix template (~5 ng/tube) was subject to PCR by using the previously determined optimal conditions. For example, when *rTaq* DNA polymerase was used, PCR was performed using Bindel-PCR(Et1)-2S/-1A primer set in the presence of 0.85 mM Mg^2+^ (Fig. 2d, right panel), whereas when HiDi DNA polymerase was used, PCR was performed using Bindel-PCR(Et1)-2S/-1A primer set in the presence of 2 mM Mg^2+^ (Fig. 2e, right panel). Both DNA polymerases yielded positive bands for samples containing the WT allele, but they failed to amplify the samples with Indel Mix 1-3, as expected (Fig. 2d,e and 3); more specifically, both DNA polymerases yielded positive bands for the samples corresponding to WT:Indel Mix = 1:1 to 1:127 (Fig. 4a). Generally, when zygotes are transfected with genome-editing components through microinjection or electroporation, genome editing at target loci is largely completed in the zygote stage or at least by the 2- or 4-cell stage^17,18^. Our results obtained using Bindel-PCR indicate that the presence of the WT allele at a target locus can discerned when mosaic mutations occur by the 2-to-4-cell stage; thus, Bindel-PCR enables identification of biallelic KO mutants among animals harbouring mosaic mutations.

### Identification of biallelic KO mutants among F0 offspring after genome editing targeting the loci *Et1*, *Tyr*, and *Ramp1* (receptor activity modifying protein-1) and among 12.5 dpc foetal samples after genome editing targeting the loci *Ramp3* and *Rosa26*

**Figure 5:**
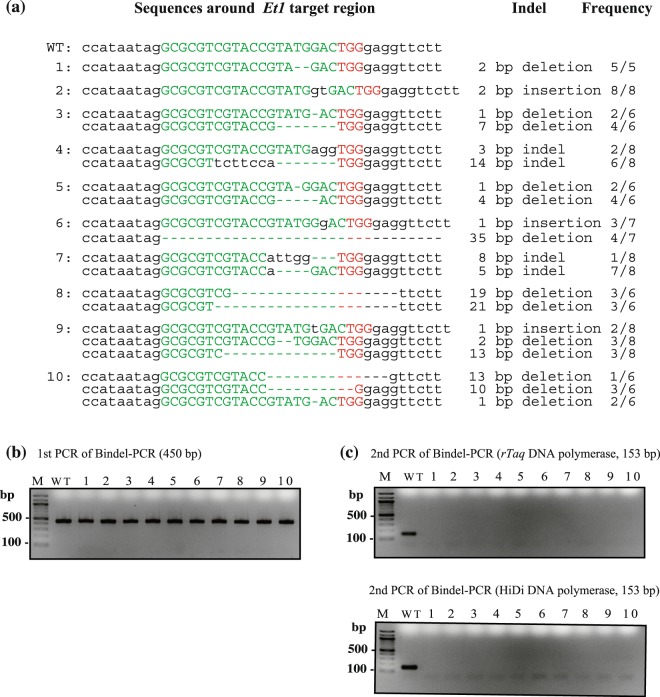
Identification of biallelic KO mutants among F0 offspring obtained after CRISPR/Cas9-mediated introduction of indels at *Et1* locus. (**a**) Sequencing results of genomic DNA samples (10) obtained from the genome-edited F0 offspring that had already been demonstrated to be biallelic KO for *Et1* locus (Sakurai *et al.*, unpublished data). Green nt correspond to the sequence recognised by gRNA-*Et1*. Red nt correspond to the PAM sequence. Dashed lines indicate nt deletion. Frequency = no. clones detected/total no. clones sequenced. (**b**) Result of 1^st^ PCR of genomic DNA samples 1–10, performed using Et1ch-2S/-2A primer set. WT, wild-type genomic DNA used as a positive control; M, 100-bp-ladder markers. (**c**) Upper panel: Results of 2^nd^ PCR performed using *rTaq* DNA polymerase and Bindel-PCR(Et1)-2S/-1A primer set in the presence of 0.85 mM MgCl_2_; lower panel: results of 2^nd^ PCR performed using HiDi DNA polymerase and Bindel-PCR(Et1)-2S/-1A primer set in the presence of 2 mM MgCl_2_. WT, wild-type genomic DNA used as a positive control; M, 100-bp-ladder markers. The images of these full-length gels are presented in Supplementary Fig. S5b,c.

As a proof-of-principle experiment to test the feasibility of Bindel-PCR, we performed 1^st^ PCR by using genomic DNA samples (10) obtained from the genome-edited F0 offspring that had already been demonstrated to be biallelic KO for *Et1* locus (Fig. 5a; Sakurai *et al*., unpublished data). After agarose-gel electrophoresis-based confirmation that the 1^st^ PCR products were successfully produced (Fig. 5b), the 2^nd^ PCR was performed using the 1^st^ PCR products as templates (Fig. 5c): With both *rTaq* and HiDi DNA polymerases, PCR products were generated from all the samples tested in the 1^st^ PCR, but almost all the samples (except for the WT samples) were judged as negative when the 2^nd^ PCR was conducted. These results indicate that Bindel-PCR is useful for rapid and convenient identification of biallelic KO mutants among F0 offspring carrying various types of indels and mosaic mutations with 100% accuracy.

**Figure 6:**
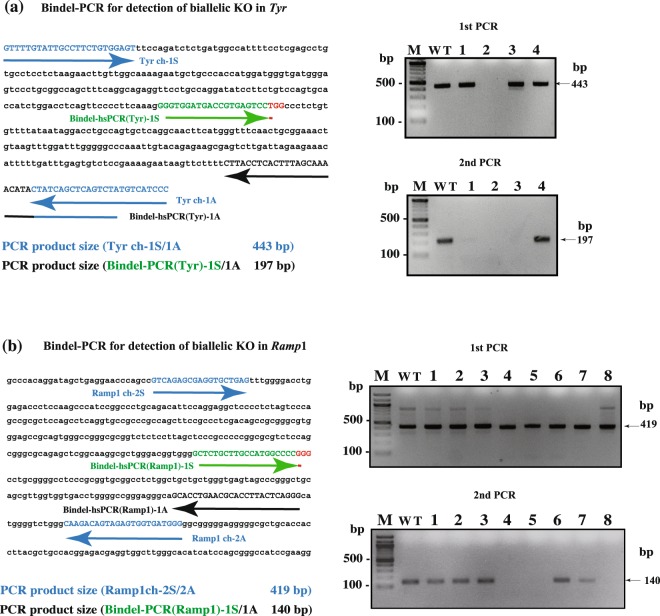
Identification of biallelic KO mutants among F0 offspring carrying CRISPR/Cas9-induced indels at *Tyr* (**a**) and *Ramp1* (**b**) loci. (**a**) Location of *Tyr* sequences recognised by gRNAs and the PCR primers used. Blue, nt sites used as forward and reverse primers for the 1st PCR; green, nt corresponding to gRNA target sequence; red, PAM sequence. Expected sizes of PCR products obtained using different primer sets are shown at the bottom. In the upper right panel, the results of the 1^st^ PCR of genomic DNA samples of three F0 mice (lanes 1–3; Supplementary Table S1) and one F1 offspring (lane 4) are shown when PCR was performed using *rTaq* DNA polymerase and Tyrch-1S/-1A primer set in the presence of 1.5 mM MgCl_2_. In the lower right panel, the results of the 2^nd^ PCR using 1^st^ PCR products are shown when PCR was performed using *rTaq* DNA polymerase and Bindel-PCR(Tyr)-1S/-1A primer set in the presence of 0.85 mM MgCl_2_. (**b**) Location of *Ramp1* sequences recognised by gRNAs and the PCR primers used. Sites indicated by blue, green, and red correspond to the sites recognised by primers, gRNA, and PAM, respectively. In the upper right panel, the results of the 1^st^ PCR of genomic DNA samples of eight F0 mice (lanes 1–8; Supplementary Table S1) are shown when PCR was performed using *rTaq* DNA polymerase and Ramp1ch-1S/-1A primer set in the presence of 1.5 mM MgCl_2_. In the lower right panel, the results of the 2^nd^ PCR of 1^st^ PCR products are shown when PCR was performed using *rTaq* DNA polymerase and Bindel-PCR(Ramp1)-1S/-1A primer set in the presence of 0.85 mM MgCl_2_. WT, wild-type genomic DNA used as a positive control; M, 100-bp-ladder markers. Images of these full-length gels are presented in Supplementary Fig. S6a,b.

We also performed *Taq* DNA polymerase-based Bindel-PCR by using F0 mice in which *Tyr* and *Ramp1* loci were targeted (Fig. 6a,b). For *Tyr*, we used 3 F0 mice (Nos 1, 2, and 3), which were obtained by electroporating C57BL/6J (B6) fertilised eggs in a solution containing *Tyr*-targeting gRNA and Cas9 protein, and one F1 offspring (No. 4) derived from mating between a B6 female and No. 1 F0 male (Fig. 6a and Supplementary Table S1). The coat colour of the 3 F0 mice was white, which indicated that they were biallelic KO for *Tyr* locus, whereas No. 4 F1 offspring was black, which indicated that it was heterozygous KO for *Tyr* locus. In the 1^st^ PCR, we used a PCR primer set (Tyrch-1S/-1A in Fig. 6a and Table S3) to amplify a portion of *Tyr* locus spanning a sequence recognised by gRNA-*Tyr* (Fig. 6a). The 1^st^ PCR thermal-cycling protocol was 95 °C for 1 min, 35 cycles of 95 °C for 15 s, 68 °C for 10 s, and 72 °C for 40 s, and the reaction was performed in a PCR buffer [10 mM Tris (pH 8.3), 50 mM KCl, 200 μM dNTPs, and 1.5 mM MgCl_2_] containing *rTaq* DNA polymerase (0.5 U/25 μL). Next, 2^nd^ PCR was performed using Bindel-PCR(Tyr)-1S/-1A primer set (Tm, ~69 °C; Fig. 6a and Table S3), and the reaction was in a solution containing the 1^st^ PCR products (0.5–1 ng) as the template; the PCR protocol was 95 °C for 1 min, 40–45 cycles of 95 °C for 15 s, 68 °C for 10 s, and 72 °C for 40 s. All tested samples (except No. 2 F0 sample) generated a 443-bp PCR product (Fig. 6a, upper right panel). Subsequent sequencing analysis revealed that No. 2 F0 sample contained an ~200-nt deletion around the target site of *Tyr* locus (Supplementary Fig. S2a). When the 2^nd^ PCR was performed, almost all samples (except No. 4 F0 and WT samples) were identified as negative (Fig. 6a, lower right panel). These results suggested that the 3 F0 mice (Nos 1–3) were biallelic KO mice, and sequencing of the samples confirmed this notion (Supplementary Fig. S2a).

For *Ramp*1, we used 8 F0 mice (Nos 1–8) (Supplementary Table S1), which had been obtained by electroporating maternal Cas9-containing sCAT fertilised eggs^19^ in a solution containing gRNA-*Ramp1* alone (Fig. 6b). The 1^st^ PCR conditions were the same as that used for checking mutations in *Et1* and *Tyr* loci. All tested samples were identified as positive (Fig. 6b, upper right panel). For 2^nd^ PCR, the 1^st^ PCR products (0.5–1 ng) were subject to nested PCR using Bindel-PCR(Ramp1)-1S/-1A primer set (Tm, 74 °C–76 °C; Fig. 6b, and Table S3) under these conditions: 95 °C for 1 min, 40–45 cycles of 95 °C for 15 s, 72 °C for 40 s, and 72 °C for 40 s. Sample Nos 4, 5, and 8 (but not WT sample) were identified as negative (Fig. 6b, lower right panel), which suggested that mice Nos. 4, 5, and 8 were biallelic KO mice; subsequent sequencing of the samples confirmed this assessment (Supplementary Fig. S2b).

Furthermore, we performed *Taq* DNA polymerase-based Bindel-PCR by using 12.5 dpc foetal samples, in which *Ramp3* and *Rosa26* loci had been targeted (Supplementary Fig. S1a,b). For detection of indels in *Ramp3*, we used 8 foetuses (nos 1–8), which were derived from C57BL/6J (B6) fertilised eggs electroporated in a solution containing *Ramp3*-targeting gRNA and Cas9 protein (Table S2 and Supplementary Fig. S1a, left drawing). The 1^st^ PCR conditions were the same as that used for checking mutations in *Et1* locus. All tested samples were identified as positive (Supplementary Fig. S1a, upper right panel). For the 2^nd^ PCR, the 1^st^ PCR products (0.5–1 ng) were subject to nested PCR by using Bindel-PCR(Ramp3)-1S/-1A primer set (Tm, 51 °C–52 °C; Supplementary Fig. S1a, left drawing and Table S3) under the following conditions: 95 °C for 1 min, 40–45 cycles of 95 °C for 15 s, 57 °C for 10 s, 72 °C for 40 s, and 72 °C for 40 s, and the reaction was performed in a PCR buffer [10 mM Tris (pH 8.3), 50 mM KCl, 200 μM dNTPs, and 0.8–0.9 mM MgCl_2_] containing *rTaq* DNA polymerase (0.5 U/25 μL). Sample numbered 5 was identified as negative (Supplementary Fig. S1a, lower right panel), suggesting that it had biallelic mutations. Subsequent sequencing of the samples confirmed this hypothesis (Supplementary Fig. S2c). Finally, for detection of indels in *Rosa26*, we used 9 foetuses (nos 1–9), which were derived from C57BL/6J (B6) fertilised eggs electroporated in a solution containing *Rosa26*-targeting gRNA and Cas9 protein (Table S2 and Supplementary Fig. S1b, left drawing). All tested samples were identified as positive (Supplementary Fig. S1b, upper right panel) as before. For the 2^nd^ PCR, the 1^st^ PCR products (0.5–1 ng) were subject to nested PCR using Bindel-PCR(Rosa26)-1S/-1A primer set (Tm, 60 °C; Supplementary Fig. S1b, left drawing and Table S3) under these conditions: 95 °C for 1 min, 40–45 cycles of 95 °C for 15 s, 68 °C for 10 s 72 °C for 40 s, and 72 °C for 40 s, and the reaction was performed in a PCR buffer [10 mM Tris (pH 8.3), 50 mM KCl, 200 μM dNTPs, and 1 mM MgCl_2_] containing *rTaq* DNA polymerase (0.5 U/25 μL). Samples numbered 6 and 9 were identified as negative (Supplementary Fig. S1b, lower right panel), suggesting that they are biallelic foetuses. This point was also confirmed by subsequent sequencing (Supplementary Fig. S2d).

## Discussion

We developed a novel method, Bindel-PCR, that enables rapid identification of biallelic indel mutants among CRISPR/Cas9-mediated genome-edited animals having indels at a region within 5 bp 5′ upstream of the PAM (where 94% or more indels are known to generate). It was demonstrated that this new technique allows rapid identification of biallelic indel mutants among samples containing various types of indels and mosaic mutations with 100% accuracy. Notably, in proof-of-principle analyses of the Bindel-PCR method, we successfully identified biallelic KO individuals carrying indels in target loci such as *Et1*, *Tyr*, *Ramp1, Ramp3* and *Rosa26*.

Bindel-PCR is characterised by the ability to detect biallelic KO samples alone among samples harbouring various types of indels through 2 PCR steps: 1^st^ PCR, for generating the PCR product that indicates successful amplification of a target region; and 2^nd^ PCR, for showing the lack of a PCR product, which indicates successful identification of biallelic mutation at a target region (Fig. 1a). This method requires only PCR primers, common enzymes for DNA amplification, and a PCR machine and agarose-gel electrophoresis system. These features distinguish our method from other detection systems that depend on the screening of mutations through entire samples by using complexed reagents, enzymatic reactions, and appropriate PCR conditions that require strict optimisation. For example, a sensitive screening technique based on conventional PCR, called ‘annealing at critical temperature PCR (ACT-PCR)’, for identifying mutants was recently reported^20^; ACT-PCR requires only a single PCR step followed by agarose-gel electrophoresis, but the critical annealing temperature should be carefully examined to avoid false-positive samples. Notably, in relation to this study, we observed that the PCR condition used under standard Mg^2+^ concentrations (1.5–2.5 mM) and an annealing step at higher temperatures close to or over Tm failed to identify biallelic KO individuals (see PCR with 1.5–2.5 mM in Fig. 2d, e for analysis of *Et1* and lower right panels of Fig. 6a,b for analysis of *Tyr*, *Ramp1*, and lower right panels of Supplementary Fig. S1a,b for analysis of *Ramp3* and *Rosa26*). Moreover, we tested various dNTP concentrations, 2-step PCR (shuttle PCR), and 3-step touchdown PCR (annealing at 72 °C to 61 °C/decreasing 1 °C per cycle), which are considered as a simple and rapid means to optimise PCR^21^, for potential establishment of Bindel-PCR, but failed (data not shown). From these analyses, we ascertained that the 3-step PCR (denature/annealing/extension) at low concentrations of MgCl_2_ is suitable for preferential selection of biallelic KO samples. PAGE has been considered useful for identifying organisms carrying indels^22^. However, detecting single-base changes such as exchange, deletion, or insertion of 1 nt remains challenging with the laboratory-standard PAGE (Fig. 3c). Furthermore, T7E1 assay is an excellent technique for identification of indels, but requires an additional *in vitro* assay (e.g., Cas9RNP cut assay) to detect biallelic KO mutations, as shown in Fig. 3b. Collectively, the present Bindel-PCR technique is more convenient than ACT-PCR, and more rapid than the pre-existing PAGE and T7E1 assay for the purpose of rapid identification of biallelic KO mutation, even though T7E1 assay appears to be superior for identifying the indels in the samples.

Interestingly, new techniques have emerged, using fluorescent-PCR or high-resolution melting-curve analysis, that facilitate rapid identification of single-nucleotide mutations^23,24^. However, the assays require expensive apparatus enabling high-resolution monitoring of the denaturation process of a double-stranded fluorescently labelled DNA fragment. The most promising approach for identifying these cells appears to be DNA sequencing of the target region showing indels by using the Sanger method or next-generation sequencing. Notably, the TIDE (Tracking of Indels by DEcomposition) method based on Sanger sequencing and its web-tool for analysis allow efficient evaluation of indel frequency^25^, but such strategies based on DNA sequencing are expensive and require several days for handling large numbers of samples.

In the present study, we estimated that the efficiency of success was >94% because the CRISPR/Cas9-induced indels spanned an area of 5 bp immediately before the PAM, and >94% of the genome-edited samples obtained thus far contained mutations in these regions (Fig. 1d); moreover, Bindel-PCR is focused on the identification of samples harbouring biallelic indels within an area of 5 bp upstream of the PAM. The other advantage of using Bindel-PCR is the simplicity of setting PCR conditions. For example, the forward PCR primer is designed to span a 20-bp sequence upstream of the PAM (NGG) or a 20-bp sequence [containing the N in the PAM (NGG)] upstream of the PAM (Figs 1a, 2a, 6 and Supplementary Fig. S1). Next, the reverse PCR primer is designed to correspond to the sequence ~200 bp downstream from the PAM. In this case, caution must be used to ensure that the Tm values of the reverse and forward primers are similar. Furthermore, the annealing temperature in the PCR performed ranged from about −3 °C to +10 °C of Tm value. Three PCR steps, featuring 40–50 cycles of 95 °C–68 °C–72 °C, were used, although Bindel-PCR directed towards *Ramp1* was performed using only 2 steps of PCR, with 40–50 cycles of 95 °C–72 °C (Fig. 6b). Optimal conditions were determined by decreasing the MgCl_2_ concentration (starting from 1.5 and 2.5 mM for *rTaq* DNA polymerase and HiDi DNA polymerase, respectively). The optimal concentration of MgCl_2_ was determined under conditions wherein PCR products from the WT samples became invisible. For detection of the biallelic KO samples in which *Et1* locus was targeted, the optimal concentration of MgCl_2_ was around 0.85 mM for *rTaq* DNA polymerase and around 2 mM for HiDi DNA polymerase. For detection of the biallelic KO samples in which *Tyr, Ramp1, Ramp3*, *and Rosa26* loci had been targeted, the optimal concentration of MgCl_2_ was around 0.85, 0.85, 0.8, and 1 mM, respectively, for *rTaq* DNA polymerase (Fig. 6 and Fig. S1). The samples that were judged as negative after PCR was performed using these MgCl_2_ concentrations are highly likely to be biallelic KO mutants.

A challenge associated with Bindel-PCR might be that biallelic mutants must be selected as PCR-negative samples. To avoid potential false judgment, we performed 1^st^ and 2^nd^ PCR, with the 2^nd^ PCR being tested in the presence of standard or low concentrations of MgCl_2_. The 1^st^ PCR was set to allow amplification of fragments spanning 400–500 bp of sequence covering the gRNA-recognising region of a target gene (Figs 2a, 6, and Supplementary Fig. S1); this PCR should yield positive PCR products (Fig. 2c, upper right panels of Fig. 6, and Supplementary Fig. S1). Furthermore, the presence of samples containing long sequence deletions in a target gene can be readily detected, because the resultant PCR products differ in size or are occasionally absent (Fig. 6a). The 2^nd^ PCR was set to allow amplification of the 1^st^ PCR products under stringent conditions achieved by reducing the MgCl_2_ concentration to detect the absence of PCR products derived from biallelic KO samples (Figs 2d,e, 5, 6, and S1). The samples showing negative results when PCR was performed under the low concentration of MgCl_2_ were judged as biallelic mutants carrying indels of ≥1 bp at least within the 5-bp sequence upstream of the PAM or indels between at least 5 bp of sequence upstream or downstream of the PAM.

Bindel-PCR allows highly sensitive detection of WT samples (Fig. 4a). This ability facilitates the detection of biallelic mutants among a group of genome-edited animals showing enrichment of mosaic mutations. Here, we examined whether Bindel-PCR can be used for effectively identifying the WT alleles included in samples enriched with mosaic mutations. For this purpose, we performed two experiments; one concerning the sensitivity of Bindel-PCR for identifying biallelic mutants among mixed samples containing various indels (Fig. 3), and the other concerning the sensitivity for detecting the presence of WT alleles in samples containing various ratios of mutant-type templates (Fig. 4). In the former, we found that Bindel-PCR could identify biallelic mutants among mixed samples containing various indels. In the latter, we prepared experimental samples, ‘WT:Indel Mix’ samples, in which genomic DNA carrying indels was mixed at various ratios with WT-mouse genomic DNA. Our results showed that Bindel-PCR performed using either *rTaq* or HiDi DNA polymerase successfully detected WT:Indel Mix = 1:127 (Fig. 4). Because mosaic mutations frequently occur at the 2-cell stage after transfection of zygotes with CRISPR/Cas9-related components, we consider Bindel-PCR to be highly effective in detecting biallelic mutants from offspring enriched with mosaic mutations. Furthermore, this high degree of sensitivity in detecting WT alleles should enable the identification of KO cells from a genome-edited polyploid cell line, such as 3T3.

Is *rTaq* or HiDi DNA polymerase optimal for Bindel-PCR? Our results support the view that both polymerases can be used for the detection of biallelic mutants. Notably, HiDi DNA polymerase was superior to *rTaq* DNA polymerase under stringent conditions: PCR product amounts were increased to a higher level with HiDi than *rTaq* DNA polymerase (Figs 2d,e and 4). Furthermore, HiDi DNA polymerase was also superior in terms of the ability to detect the WT samples among samples enriched with mosaic mutations (Fig. 4). In this context, it might be appropriate to select the DNA polymerase according to the complexity of the genome-edited samples to be examined.

In summary, Bindel-PCR is a *T. aquaticus* DNA polymerase-based PCR system that enables rapid detection of biallelic KO mutants in CRISPR/Cas9-mediated genome-edited organisms, and this PCR system requires no special reagents or apparatus. Given its simplicity and sensitivity, we expect Bindel-PCR to serve as an effective screening tool for identifying biallelic KO cells/individuals from large numbers of samples.

## Methods

### Ethics approval of mouse experiments

All experimental animal procedures were performed according to the guidelines of *Shinshu University Committee* on *Recombinant DNA Security* and were approved by *the Animal Care and Experimentation Committee of Shinshu University* (permit no. 300044).

### Mice

C57BL/6J (hereafter ‘B6’) and ICR mice were purchased from CLEA Japan Inc. (Tokyo, Japan). Systemic Cas9-expressing Tg mice (‘sCAT’ mice) were produced in-house^19^.

### Data source for nucleotide sites frequently showing CRISPR/Cas9-induced indels

To investigate the sites that are susceptible to CRISPR/Cas9-induced mutation, we used the data obtained in our recent study (Sakurai *et al*., unpublished data) for mouse *Et1* and in other studies for rat *Tyr*, *Rosa26*, *Sirpa*, and *Cyp2d*^15^, and for mouse *Fgf10*^11^.

### Reagents and PCR primers

Reagents were purchased from Wako Pure Chemical Industries, (Osaka, Japan), unless specified otherwise. The primers used for PCR are listed in Supplementary Table S3; the primers were ordered for synthesis as desalted oligonucleotides (Invitrogen Custom Primers Japan, Thermo Fisher Scientific K.K., Tokyo, Japan).

### PCR, T7E1 assay, and gel image acquisition

We used 3 DNA polymerases in this study. For constructing a series of *Et1* plasmids carrying indels, we used *Tks Gflex* DNA polymerase, a high-fidelity DNA polymerase derived from *Thermococcus* sp. (TaKaRa Bio. Inc., Shiga, Japan). For the establishment of Bindel-PCR and for its fidelity test, we used a cloned *Taq* (*rTaq*) DNA polymerase derived from *T. aquaticus* DNA polymerase (TaKaRa Taq^TM^ #R001A, TaKaRa) and HiDi DNA polymerase (myPOLS Biotec #9001, myPOLS Biotec GmbH, Konstanz, Germany). We prepared MgCl_2_-free PCR reaction buffers for *rTaq* DNA polymerase [10×; 100 mM Tris (pH 8.3), 500 mM KCl, and 2 mM dNTPs] and HiDi DNA polymerase [10×; 500 mM Tris (pH 9.2), 160 mM (NH_4_)_2_SO_4_, 1% Tween-20, and 2 mM dNTPs], and before PCR, we added MgCl_2_ at appropriate concentrations to the PCR reaction buffer (1×). PCR was performed in a 25-μL volume, and 10-μL of the PCR products were electrophoresed using a 1.5% agarose-gel/TAE buffer system and a standard 5% polyacrylamide gel/TBE buffer system used in the laboratory. T7E1 assay was performed as described by Sakurai *et al*.^12^. The Gel images were acquired by 0.3 s exposure time with a CCD-gel imaging system (Nippon Genetics FAS-VI, Nippon Genetics, Tokyo, Japan) and cropped by Adobe Photoshop CS5 (12.0.4 × 64 version, Adobe Systems, CA, USA).

### *Et1* plasmids carrying a series of indels

We generated a series of *Et1* plasmids carrying various types of indels (Fig. 2b). First, we constructed an *Et1* plasmid carrying WT *Et1* cDNA. Genomic DNA isolated from a B6 mouse was PCR-amplified using an Et1ch-2S/-2A primer set to obtain a 450-bp PCR product, which was then subcloned into pBluescript II (Agilent Technologies Japan, Tokyo, Japan) to obtain pBs/WT *Et1*. The resultant plasmid was sequenced to confirm that the inserted fragment was the authentic cDNA. Next, we generated a series of *Et1* plasmids harbouring indels through inverse-PCR performed using pBs/WT *Et1* as template, and the resultant *Et1* plasmids were sequenced to confirm that they contained the appropriate target sequences.

### Preparation of model samples used for fidelity test of Bindel-PCR

For testing the fidelity of Bindel-PCR, we used samples carrying indels in the loci *Et1* (Fig. 5), *Tyr*, *Ramp1*, *Ramp3* and *Rosa26* (Figs 6, S1, S2, and Supplementary Tables S1 and S2). For generating mice harbouring indels, we employed the *in vitro* zygote electroporation method^11^ using CUY21Edit II (BEX, Tokyo, Japan). The gRNAs targeting *Tyr, Ramp1, Ramp3*, and *Rosa26* (Fig. 6 and Supplementary Fig. S1) were prepared as described by Sakurai *et al*.^12^, and the zygotes were prepared according to the methods described in our previous study^19^. For producing mice harbouring indels in *Tyr*, *Ramp3* and *Rosa26* loci, we used B6-derived zygotes and a *Tyr, Ramp3* and *Rosa26*-targeting gRNA complexed with Cas9 protein (IDT #1074181, IDT Inc., IL, USA). For generating mice carrying indels in *Ramp1* locus, we used sCAT zygotes^19^ and a *Ramp1*-targeting gRNA alone. Zygotes electroporated *in vitro* in the presence of CRISPR/Cas9 components were transferred to the oviducts of pseudopregnant ICR females, which then delivered the pups and 12.5 dpc foetuses. For indel analysis, genomic DNA was isolated from a piece of ear dissected from the pups that were born, and 12.5 dpc foetuses, and the region recognised by the gRNAs was PCR-amplified. Subsequently, the resultant PCR products were subcloned into a TA cloning vector (TaKaRa). Sequencing was performed using BigDye Terminator Cycle Sequencing Kit ver3.1 and an ABI Genetic Analyzer 3130 (Applied Biosystems, Life Technologies Japan, Tokyo, Japan). Data were analysed using Genetyx-Mac ver.13.0.3 (Software Development, Tokyo, Japan) and ClustalW server (http://www.genome.jp/tools-bin/clustalw).

## Supplementary information

Supplementary Information
