# Supplementary material for: Bindel-PCR: a novel and convenient method for identifying CRISPR/Cas9-induced biallelic mutants through modified PCR using Thermus aquaticus DNA polymerase
Source: Sci Rep. 2019 Jul 9;9:9923. doi: 10.1038/s41598-019-46357-8 (PMC6617447; doi:10.1038/s41598-019-46357-8)

Supplementary Information for

Scientific Reports

Bindel-PCR: a novel and convenient method for identifying CRISPR/Cas9-induced biallelic mutants through modified PCR using *Thermus aquaticus* DNA polymerase

Takayuki Sakurai[1, 2, *], Akiko Kamiyoshi[1, 2], Norio Takei[3], Satoshi Watanabe[4], Masahiro Sato[5] and Takayuki Shindo[1, 2]

[1]Department of Life Innovation, Institute for Biomedical Sciences, Shinshu University, 3-1-1 Asahi, Matsumoto, Nagano 390-8621, Japan

[2]Department of Cardiovascular Research, School of Medicine, Shinshu University, 3-1-1 Asahi, Matsumoto, Nagano 390-8621, Japan

[3]Department of Molecular Therapeutics, Center for Food and Medical Innovation, Institute for the Promotion of Business-Regional Collaboration, Hokkaido University, Kita-21 Nishi-11, Kita-ku, Sapporo 001-0021, Japan

[4]Animal Genome Research Unit, Division of Animal Science, National Institute of Agrobiological Sciences, Ibaraki 305-8602, Japan

[5]Section of Gene Expression Regulation, Frontier Science Research Center, Kagoshima University, 8-35-1 Sakuragaoka, Kagoshima, Kagoshima 890-8544, Japan

*** Correspondence: e-mail: tsakurai@shinshu-u.ac.jp**

Supplementary Figs. S1 to S2

Supplementary Tables S1 to S3

The full-length gel data

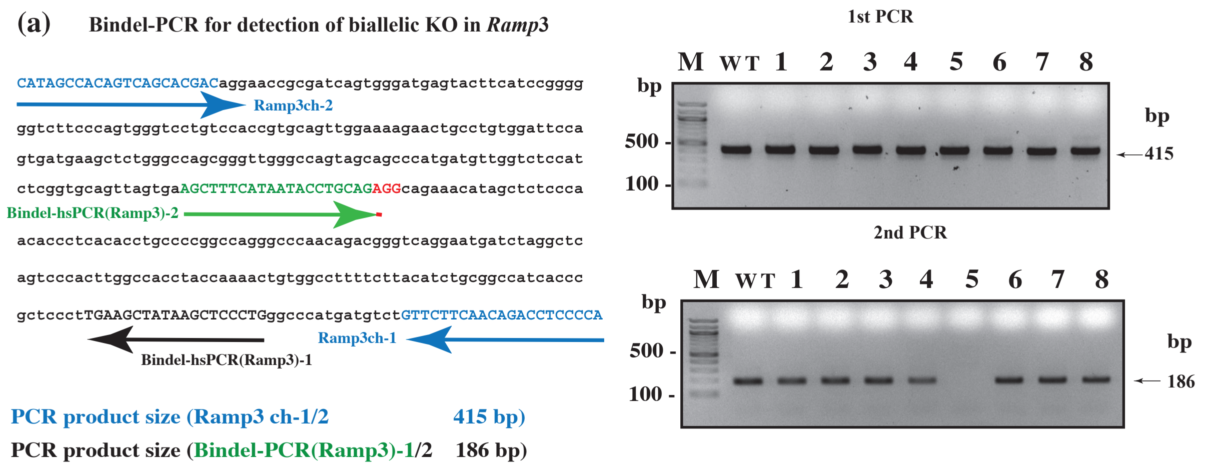
(a) Bindel-PCR for detection of biallelic KO in Ramp3
CATAGCCACAGTCAGCACGACaggaaccgcgatcagtgggatgagtacttcatccgggg
Ramp3ch-2
ggtcttcccagtgggtcctgtccaccgtgcagttggaaaagaactgcctgtggattcca
gtgatgaagctctgggccagcgggttgggccagtagcagcccatgatgttggtctccat
ctcggtgcagttagtgaAGCTTTCATAATACCTGCAGAGGcagaaacatagctctccca
Bindel-hsPCR(Ramp3)-2
acaccctcacacctgccccggccagggcccaacagacgggtcaggaatgatctaggctc
agtcccacttggccacctaccaaaactgtggccttttcttacatctgcggccatcaccc
gctccctTGAAGCTATAAGCTCCCTGggcccatgatgtctGTTCTTCAACAGACCTCCCCA
Ramp3ch-1
Bindel-hsPCR(Ramp3)-1
PCR product size (Ramp3 ch-1/2 415 bp)
PCR product size (Bindel-PCR(Ramp3)-1/2 186 bp)
1st PCR
M WT 1 2 3 4 5 6 7 8
bp
500 -
100 -
bp
415
2nd PCR
M WT 1 2 3 4 5 6 7 8
bp
500 -
100 -
bp
186

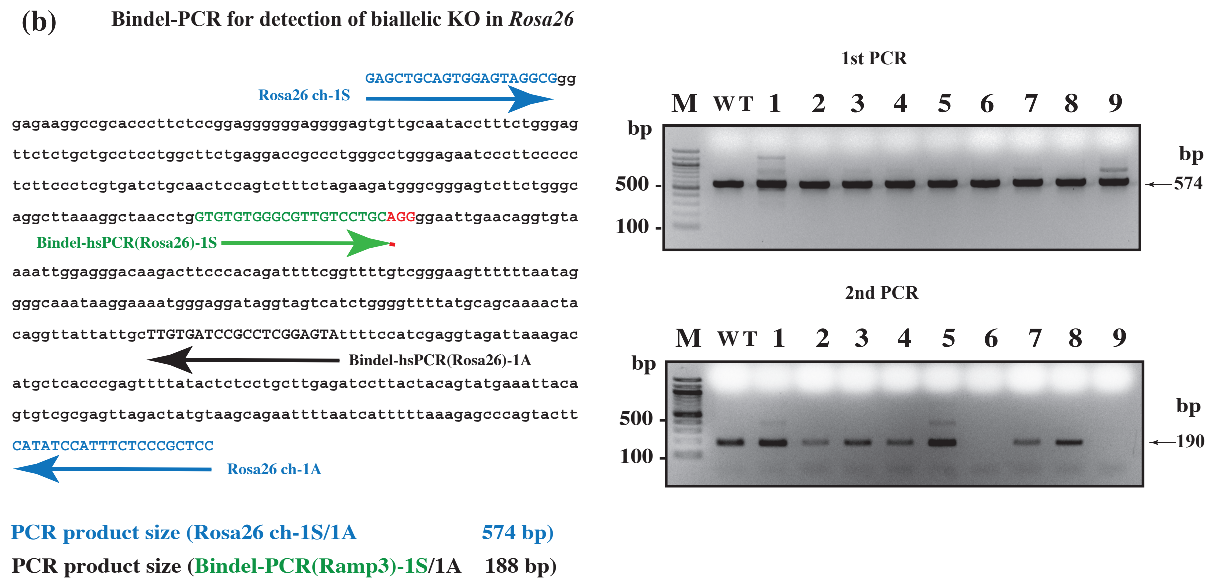
(b) Bindel-PCR for detection of biallelic KO in Rosa26
GAGCTGCAGTGGAGTAGGCGgg
Rosa26 ch-1S
gagaaggccgcacccttctccggagggggagggggagtgttgcaatacctttctgggag
ttctctgctgcctcctggcttctgaggaccgccctgggcctgggagaatcccttccccc
tcttccctcgtgatctgcaactccagtctttctagaagatgggcgggagtcttctgggc
aggcttaaaggctaacctgGTGTGTGGGCGTTGTCCTGCAGGggaattgaacaggtgta
Bindel-hsPCR(Rosa26)-1S
aaattggagggacaagacttcccacagattttcggttttgtcgggaagtttttttaatag
gggcaaataaggaaaatgggaggataggtagtcatctggggttttatgcagcaaaacta
caggttattattgcTTGTGATCCGCCTCGGAGTAtttccatcgaggtagattaaagac
Bindel-hsPCR(Rosa26)-1A
atgctcacccgagttttatactctcctgcttgagatccttactacagtatgaaattaca
gtgtcgcgagttagactatgtaagcagaattttaatcatttttaaagagcccagtactt
CATATCCATTTCTCCCGCTCC
Rosa26 ch-1A
PCR product size (Rosa26 ch-1S/1A 574 bp)
PCR product size (Bindel-PCR(Ramp3)-1S/1A 188 bp)
1st PCR
M WT 1 2 3 4 5 6 7 8 9
bp
500 -
100 -
bp
574
2nd PCR
M WT 1 2 3 4 5 6 7 8 9
bp
500 -
100 -
bp
190

Figure S1. Identification of biallelic KO mutants among 12.5 dpc foetuses carrying CRISPR/Cas9-induced indels at Ramp3 (a) and Rosa26 (b) loci. (a) Location of Ramp3 sequences recognised by gRNAs and the PCR primers used. Blue: nt sites used as forward and reverse primers for 1st PCR; green: nt corresponding to gRNA target sequence; red: PAM sequence. Expected sizes of PCR products obtained using different primer sets are shown at the bottom. In the upper right panel, the results of the 1st PCR of genomic DNA samples of eight foetuses (lanes 1–8; Supplementary Table S2) are shown when PCR was performed using rTaq DNA polymerase and Ramp3ch-1S/-1A primer set in the presence of 1.5 mM MgCl2. In the lower right panel, the results of the 2nd PCR of the 1st PCR products are shown when PCR was performed using rTaq DNA polymerase and Bindel-PCR(Ramp3)-1S/-1A primer set in the presence of 0.8 mM MgCl2. (b) Location of Rosa26 sequences recognised by gRNAs and the PCR primers used. Sites indicated by blue, green, and red correspond to the sites recognised by primers, gRNA, and PAM, respectively. In the upper right panel, the results of the 1st PCR of genomic DNA samples of nine foetuses (lanes 1–9; Supplementary Table S2) are shown when PCR was performed using rTaq DNA polymerase and Rosa26ch-1S/-1A primer set in the presence of 1.5 mM MgCl2. In the lower right panel, the results of the 2nd PCR of the 1st PCR products are shown when PCR was performed using rTaq DNA polymerase and Bindel-PCR(Rosa26)-1S/-1A primer set in the presence of 1 mM MgCl2. WT, wild-type genomic DNA used as a positive control; M, 100-bp-ladder markers. Images of these full-length gels are presented in Supplementary Fig. S1a and S1b.

(a)

	Sequences around *Tyr* target region	Indel	Frequency
WT:	ccttcaaagGGGTGGATGACCGTGAGTCCTGGccctctgtgttttataataggacc		
1:	ccttcaaagGGGTGGATGACCGTGA----TGGccctctgtgttttataataggacc	4 bp deletion	1/10
	ccttcaaagGGGTGGATGACacaga----TGGccctctgtgttttataataggacc	9 bp indel	4/10
	ccttcaaagGGGTGGATGACC--------TGGccctctgtgttttataataggacc	8 bp deletion	5/10
2:	--	~200 bp deletion	ND
3:	ccttcaaagGGG--	71 bp indel	4/10
	ccttcaaagGGGTGGAT-------------GGccctctgtgttttataataggacc	13 bp deletion	6/10
4:	ccttcaaagGGGTGGATGACCGTGA----TGGccctctgtgttttataataggacc	4 bp deletion	6/10
	ccttcaaagGGGTGGATGACCGTGAGTCCTGGccctctgtgttttataataggacc	Intact sequence	4/10

(b)

	Sequences around *Ramp1* target region	Indel	Frequency
WT:	gacggtgggGCTCTGCTTGCCATGGCCCCGGGcctgcggggcctcccgcggtgcgg		
1:	gacggtgggGCTCTGCTTGCCAagat----GGcctgcggggcctcccgcggtgcgg	8 bp indel	2/8
	gacggtgggGCTCTGCTTGCCATGGCCCCGGGcctgcggggcctcccgcggtgcgg	Intact sequence	6/8
2:	gacggtgggGCTCTGCTTGCCATGGCCCCGGGcctgcggggcctcccgcggtgcgg	Intact sequence	8/8
3:	gacggtgggGCTCTGCTTGCCATGGCCCCGGGcctgcggggcctcccgcggtgcgg	Intact sequence	8/8
4:	gacggtgggGCTCTGCTTGCCATGtcat---------------tcccgcggtgcgg	19 bp deletion	2/8
	gacggtgggGCTCTGCTT-----------------------------gcggtgcgg	29 bp deletion	6/8
5:	gacggtgggGCTCTGCTTGCCATG-----------------------gcggtgcgg	23 bp deletion	8/8
6:	gacggtgggGCTCTGCTTGCCATGGCCCCGGGcctgcggggcctcccgcggtgcgg	Intact sequence	8/8
7:	gacggtgggGCTCTGCTT-----------------------------gcggtgcgg	29 bp deletion	2/8
	gacggtgggGCTCTGCTTGCCATG-------Gcctgcggggcctcccgcggtgcgg	7 bp deletion	4/8
	gacggtgggGCTCTGCTTGCCATGGCCCCGGGcctgcggggcctcccgcggtgcgg	Intact sequence	2/8
8:	gacggtgggGCTCTGCTTGCCcg---CCCGGGcctgcggggcctcccgcggtgcgg	5 pb indel	2/6
	gacggtgggGCTCT--	47 bp deletion	4/6

(c)

	Sequences around *Ramp3* target region	Indel	Frequency
WT:	cagttagtgaAGCTTTCATAATACCTGCAGAGGcagaaacatagctctcccaacaccc		
1:	cagttagtgaAGCTTTCATAATACCTaaGCAGAGGcagaaacatagctctcccaacaccc	2 bp insertion	1/6
	cagttagtgaAGCTTTCATAATACC---AGAGGcagaaacatagctctcccaacaccc	3 bp deletion	3/6
	cagttagtgaAGCTTTCATAATACCTGCAGAGGcagaaacatagctctcccaacaccc	Intact sequence	2/6
2:	cagttagtgaAGCTTTCATAATACCTG-AGAGGcagaaacatagctctcccaacaccc	1 bp deletion	1/6
	cagttagtgaAGCTTTCATAATACCTGCAGAGGcagaaacatagctctcccaacaccc	Intact sequence	5/6
3:	cagttagtgaAGCTTTCATAATACC---AGAGGcagaaacatagctctcccaacaccc	3 bp deletion	2/6
	cagttagtgaAGCTTTCATAATACCTGCAGAGGcagaaacatagctctcccaacaccc	Intact sequence	4/6
4:	cagttagtgaAGCTTTCATAATACC---AGAGGcagaaacatagctctcccaacaccc	3 bp deletion	4/6
	cagttagtgaAGCTTTCATAATACCTGCAGAGGcagaaacatagctctcccaacaccc	Intact sequence	2/6
5:	cagttagtgaAGCTTTCATAATACC---AGAGGcagaaacatagctctcccaacaccc	3 bp deletion	4/6
	cagttagtgaAGCTTTCATAATA---------Gcagaaacatagctctcccaacaccc	9 bp deletion	2/6
6:	cagttagtgaAGCTTTCATAATACCTGCAGAGGcagaaacatagctctcccaacaccc	Intact sequence	6/6
7:	cagttagtgaAGCTTTCATAATA----------------catagctctcccaacaccc	16 bp deletion	1/6
	cagttagtgaAGCTTTCATAATACCTGCAGAGGcagaaacatagctctcccaacaccc	Intact sequence	5/6
8:	cagttagtgaAGCTTTCATAATACC--CAGAGGcagaaacatagctctcccaacaccc	2 bp deletion	3/6
	cagttagtgaAGCTTTCATAATACCTGCAGAGGcagaaacatagctctcccaacaccc	Intact sequence	3/6

(d)

	Sequences around *Rosa26* target region	Indel	Frequency
WT:	ggctaacctgGTGTGTGGGCGTTGTCCTGCAGGggaattgaacaggtgtaaaattgga		
1:	ggctaacctgGTGTGTGGGCGTTG-----CAGGggaattgaacaggtgtaaaattgga	5 bp deletion	1/6
	aacagaactcgtcctgttcattcaaccatgAGGggaattgaacaggtgtaaaattgga	42 bp insertion	3/6
	ggctaacctgGTGTGTGGGCGTTGTCCTGCAGGggaattgaacaggtgtaaaattgga	Intact sequence	2/6
2:	ggctaacctgGTGTGTGGGCGTTG-----CAGGggaattgaacaggtgtaaaattgga	5 bp deletion	1/6
	ggctaacctgGTGTGTGGGCGTTG---TGCAGGggaattgaacaggtgtaaaattgga	3 bp deletion	3/6
	ggctaacctgGTGTGTGGGCGTTGTCCTGCAGGggaattgaacaggtgtaaaattgga	Intact sequence	2/6
3:	ggctaacctgGTGTGTGGG---------------gaattgaacaggtgtaaaattgga	15 bp deletion	1/6
	ggctaacctgGTGTGTGGGCGTTGTCCTGCAGGggaattgaacaggtgtaaaattgga	Intact sequence	5/6
4:	ggctaacctgGTGTGTGGGCGTTG-CCTGCAGGggaattgaacaggtgtaaaattgga	1 bp deletion	2/6
	ggctaacctgGTGTG----------------GGggaattgaacaggtgtaaaattgga	16 bp deletion	2/6
	ggctaacctgGTGTGTGGGCGTTGTCCTGCAGGggaattgaacaggtgtaaaattgga	Intact sequence	2/6
5:	ggctaacctgGTGTGTGGGCGTTG---TGCAGGggaattgaacaggtgtaaaattgga	3 bp deletion	5/6
	ggctaacctgGTGTGTGGGCGTTGTCCTGCAGGggaattgaacaggtgtaaaattgga	Intact sequence	1/6
6:	ggctaacctgGTGTGTGGGCGTTG-----CAGGggaattgaacaggtgtaaaattgga	5 bp deletion	1/6
	ggctaacctgGTGTGTGGGCGTTGTCa-----Gggaattgaacaggtgtaaaattgga	6 bp indel	5/6
7:	ggctaacctgGTGTGTGGGCGTTG-----CAGGggaattgaacaggtgtaaaattgga	5 bp deletion	1/6
	ggctaacctgGTGTGTG----------TGCAGGggaattgaacaggtgtaaaattgga	10 bp deletion	2/6
	ggctaacctgGTGTGTGG----------GCAGGggaattgaacaggtgtaaaattgga	10 bp deletion	2/6
	ggctaacctgGTGTGTGGGCGTTGTCCTGCAGGggaattgaacaggtgtaaaattgga	Intact sequence	1/6
8:	ggctaacctgGTGTGTGGGCGTTGTCCTGCAGGggaattgaacaggtgtaaaattgga	Intact sequence	6/6
9:	ggctaacctgGTGTGTGGGCGTTGTCC------ggaattgaacaggtgtaaaattgga	6 bp deletion	1/6
	ggctaacctgGTGTGTGGGCGTTGTt-TGCAGGggaattgaacaggtgtaaaattgga	2 bp indel	2/6
	ggctaacctgGTGTGTGGGCGTTG-----CAGGggaattgaacaggtgtaaaattgga	5 bp deletion	3/6

Figure S2. Sequencing analyses of F0 mice targeted at Tyr and Ramp1 loci. Sequencing results of the 1st PCR products from F0 mice: (a) nos. 1, 3, and 4, mice targeted at Tyr locus; (b) nos. 1–8, mice targeted at Ramp1 locus; (c) nos. 1–8, mice targeted at Ramp3 locus; and (d) nos. 1–9, mice targeted at Rosa26 locus. Green, nt corresponding to gRNA target sequence; red, PAM sequence. Frequency = number of clones detected/total number of clones sequenced.

Supplementary Tables

Table S1. Creation of F0 mice by CRISPR/Cas9-based genome editing.

Target gene	Strain	CRISPR reagents	No. zygotes transferred	No. pups obtained	Total pups analyzed	Note
Tyr	B6	*Tyr* gRNA<br>Cas 9 protein	60	3	3	These three mice had white-coat color
Ramp1	sCAT	*Ramp1*gRNA<br>Maternal Cas9	52	20	8	

Table S2. Creation of 12.5dpc fetus by CRISPR/Cas9-based genome editing.

Target gene	Strain	CRISPR reagents	No. zygotes transferred	No. fetus obtained	Total fetus analyzed
Ramp3	B6	*Ramp3* gRNA<br>Cas 9 protein	30	8	8
Rosa26	B6	*Rosa26* gRNA<br>Cas9 protein	30	9	9

Table S3. PCR primers used in this study.

ID	Sequence (5' - 3')
Bindel-PCR(Et1)-1S	GCGCGTCGTACCGTATGGAC
Bindel-PCR(Et1)-2S	CGCGTCGTACCGTATGGACT
Bindel-PCR(Et1)-1A	GTTCTTTTCCTGCTTGGCAGAAATTC
Bindel-PCR(Ramp1)-1S	CTCTGCTTGCCATGGCCCCG
Bindel-PCR(Ramp1)-1A	GCACCTGAACGCACCTTACTCAGGG
Bindel-PCR(Ramp3)-1	CAGGGAGCTTATAGCTTCA
Bindel-PCR(Ramp3)-2	AGCTTTCATAATACCTGCAGA
Bindel-PCR(Rosa26)-1S	TGTGGGCGTTGTCCTGCA
Bindel-PCR(Rosa26)-1A	TACTCCGAGGCGGATCACAAG
Bindel-PCR(Tyr)-1S	GGGTGGATGACCGTGAGTCC
Bindel-PCR(Tyr)-1A	GAGCTGATAGTATGTTTTGCTAAAGTGAGGTAAG
Et1ch-2S	TACAAAGCAGAAGCCCAAACAGAGGTTG
Et1ch-2A	GTTCTTTTCCTGCTTGGCAGAAATTCCA
Ramp1ch-2S	GTCAGAGCGAGGTGCTGAG
Ramp1ch-2A	CAAGACAGTAGAGTGGTGATGGG
Ramp3ch-1	TGGGGAGGTCTGTTGAAGAAC
Ramp3ch-2	CATAGCCACAGTCAGCACGAC
Rosa26ch-1S	GAGCTGCAGTGGAGTAGGCG
Rosa26ch-1A	GGAGCGGGAGAAATGGATATGAAG
Tyrch-1S	GTTTTGTATTGCCTTCTGTGGAGT
Tyrch-1A	GGGATGACATAGACTGAGCTGATAG

The full-length gel data

Supplementary Information
Figure 2c

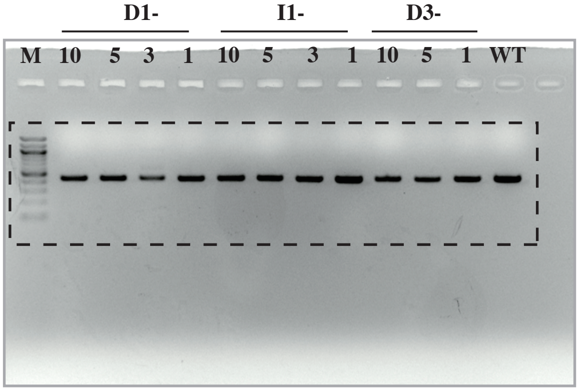

Supplementary Information
Figure 2d

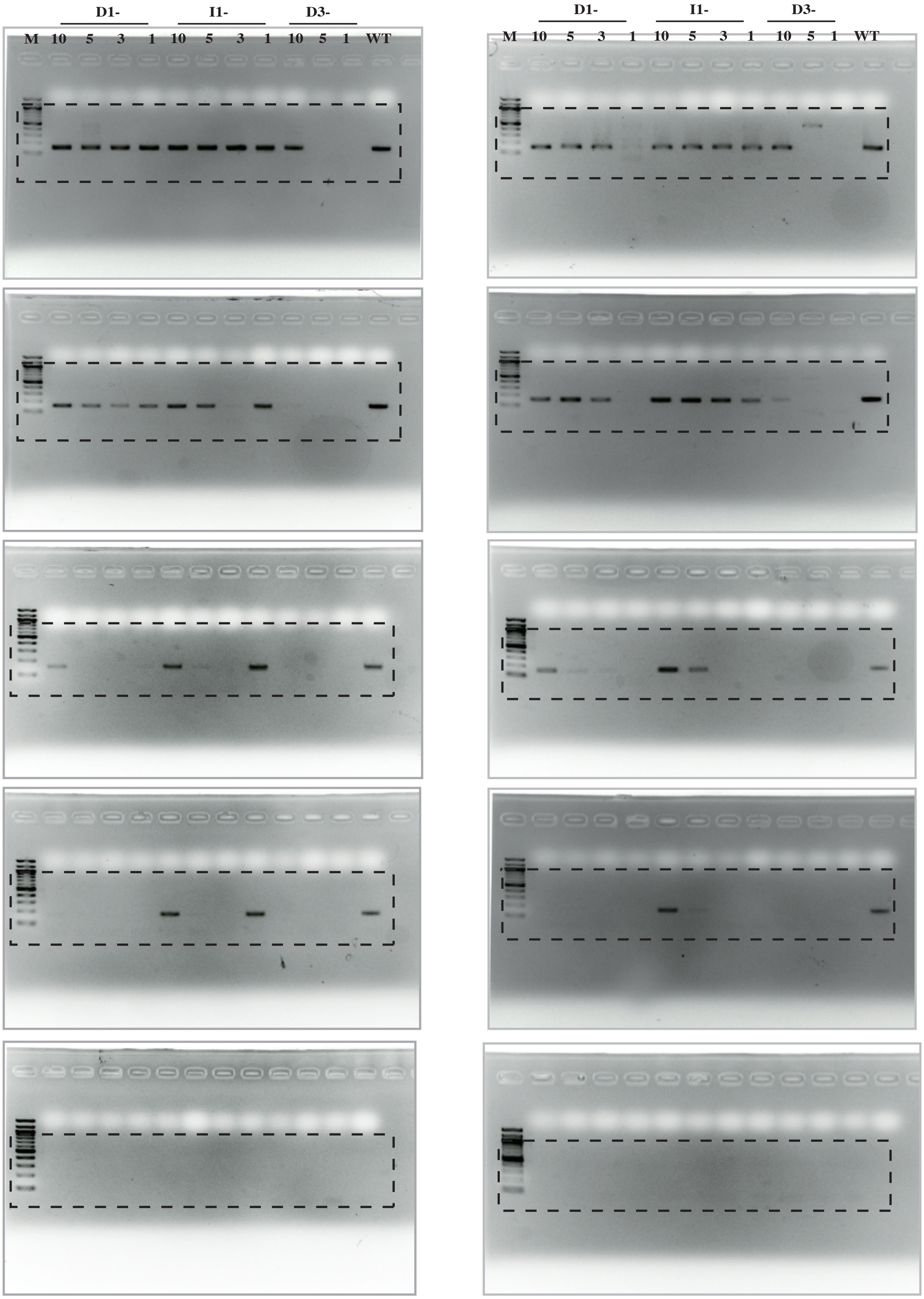

Supplementary Information
Figure 2e

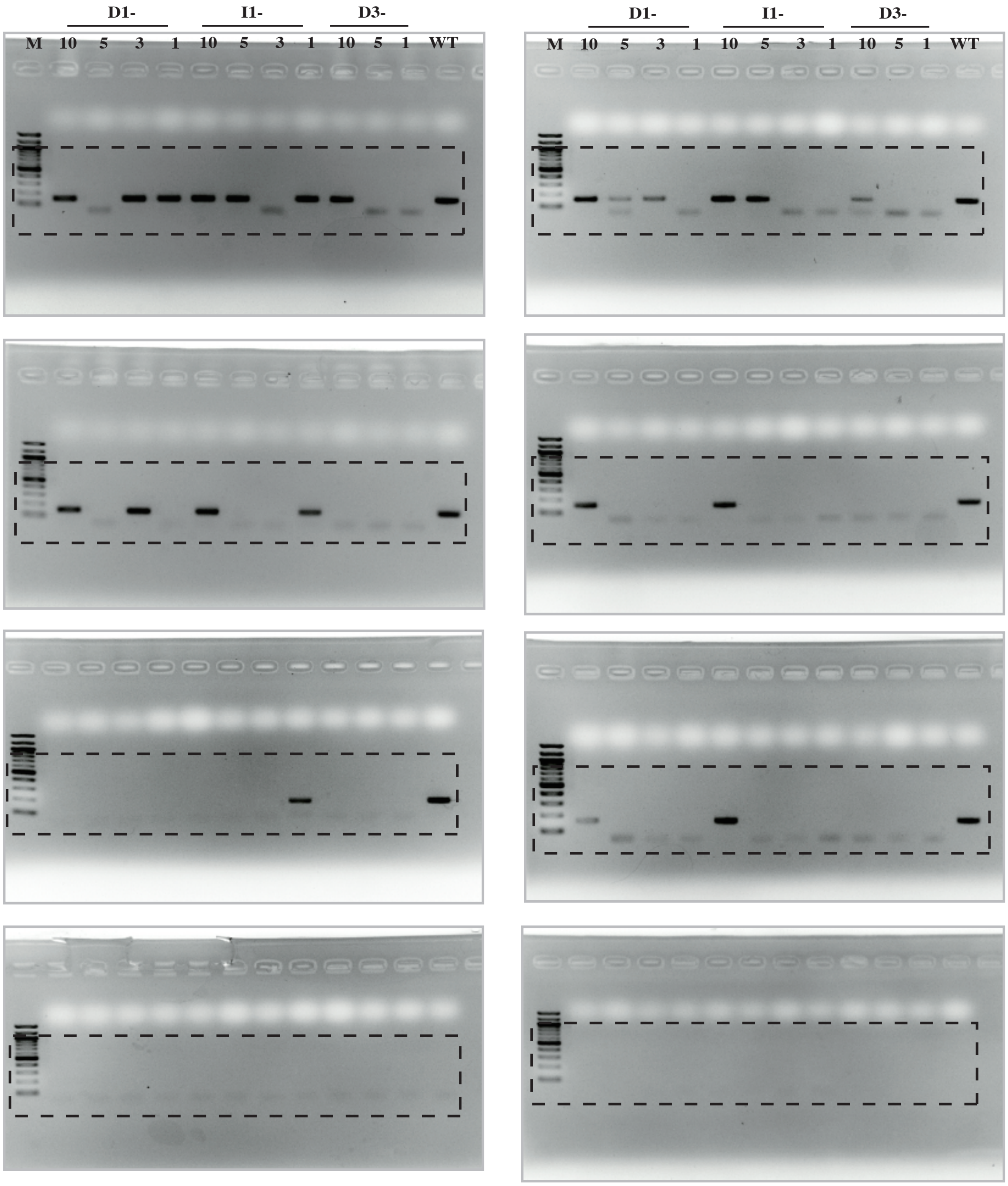

Supplementary Information

Figure 3a

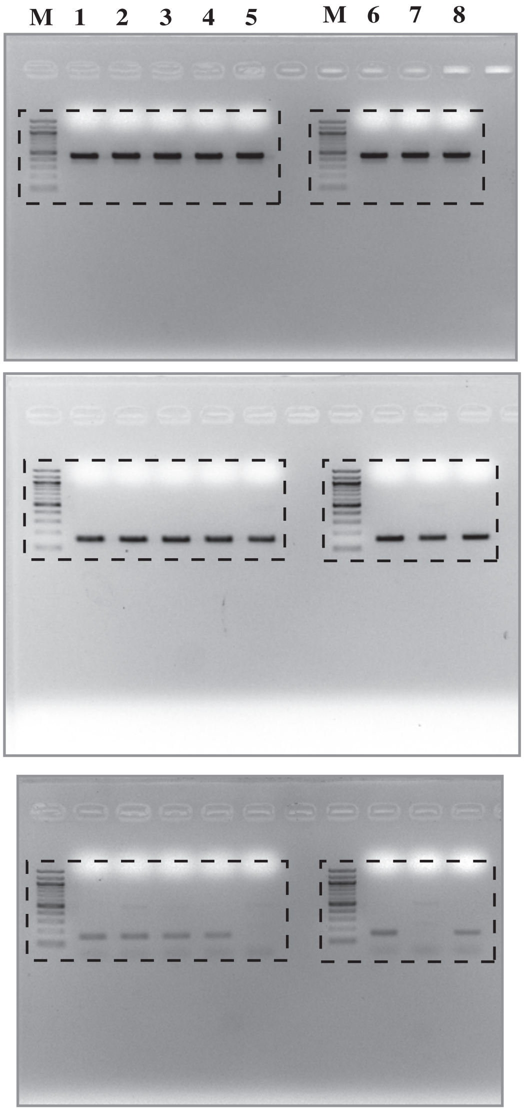

Figure 3b

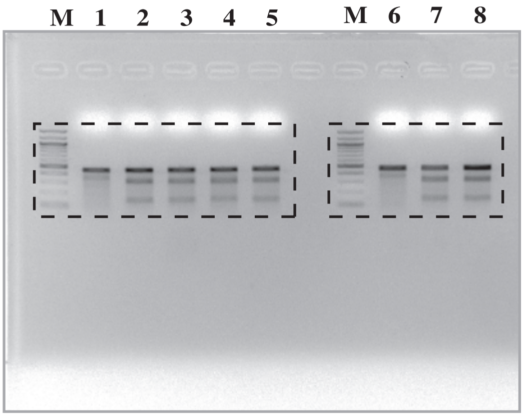

Figure 3c

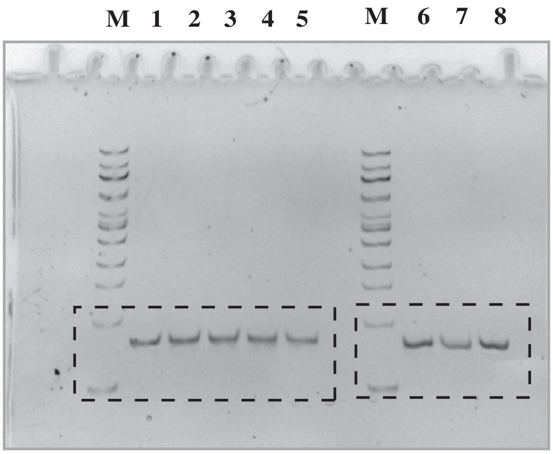

Supplementary Information
Figure 4a

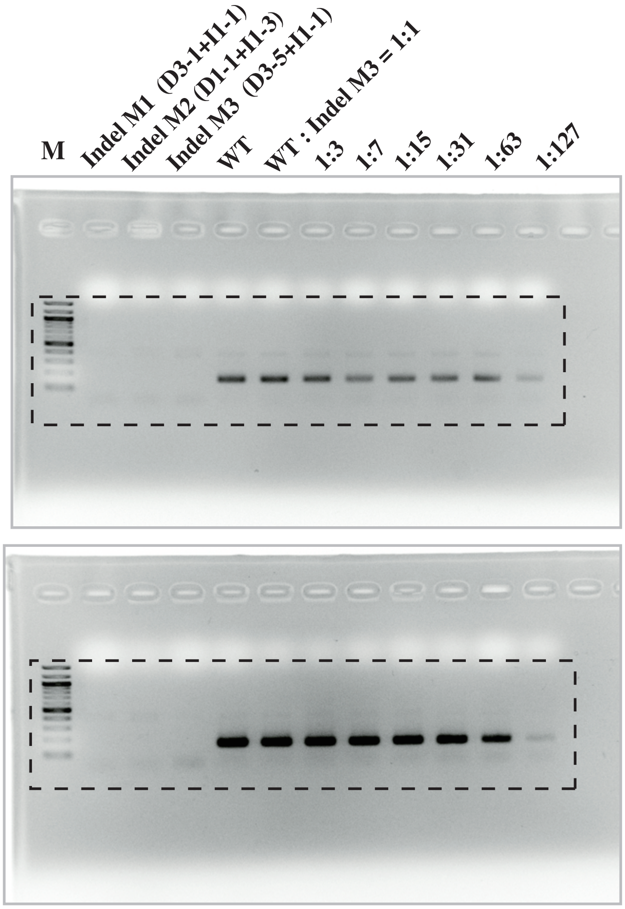

Supplementary Information

Figure 5b

M WT 1 2 3 4 5 6 7 8 9 10

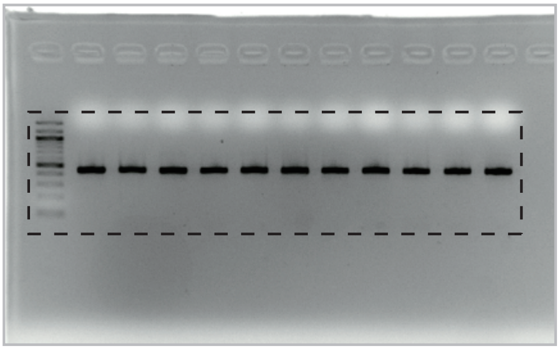

Figure 5c

M WT 1 2 3 4 5 6 7 8 9 10

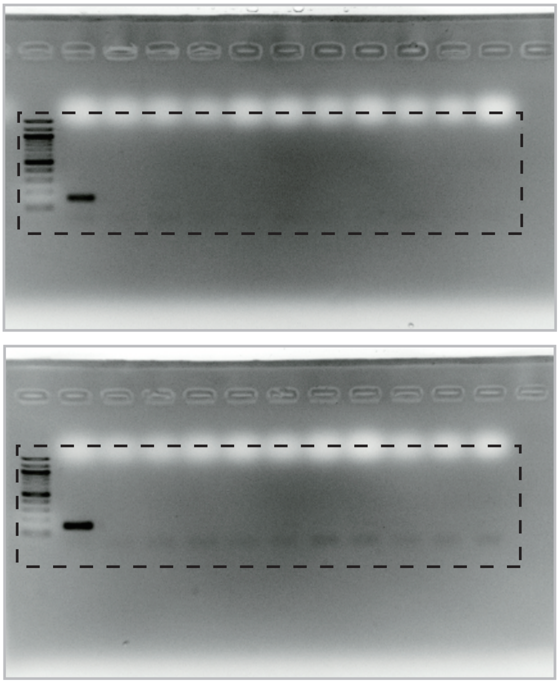

Supplementary Information

Figure 6a

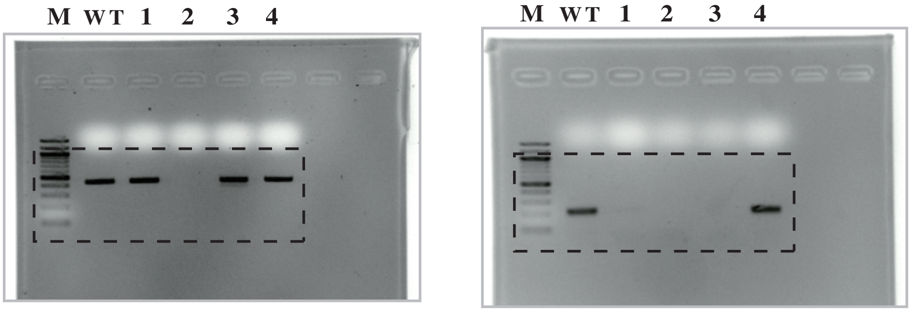

Figure 6b

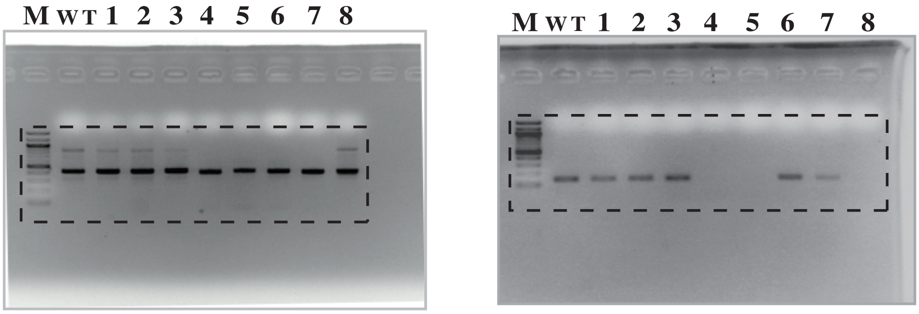

Supplementary Information

Figure S1a

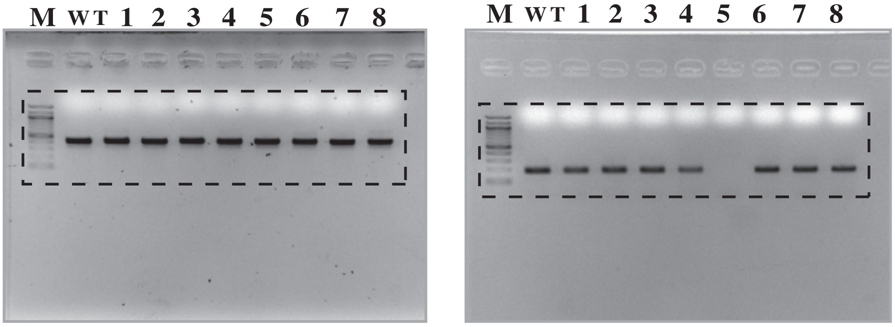

Figure S1b

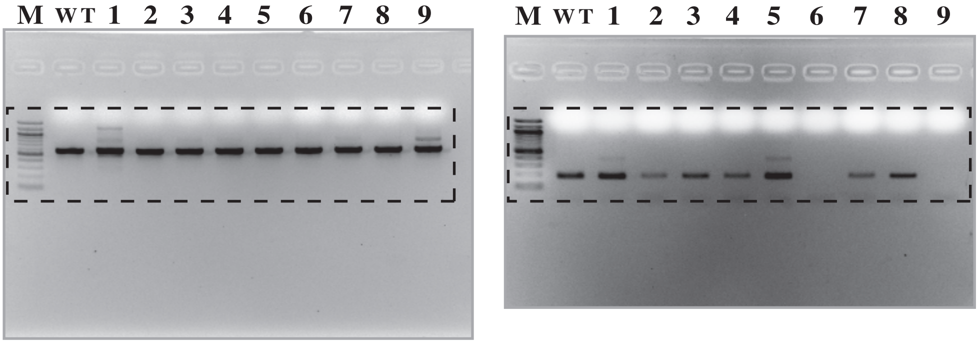

Supplement: Supplementary file 1 — Supplementary Information [file 41598_2019_46357_MOESM1_ESM.pdf]
